# Peripheral blood RNA biomarkers for cardiovascular disease from bench to bedside: a position paper from the EU-CardioRNA COST action CA17129

**DOI:** 10.1093/cvr/cvab327

**Published:** 2021-10-14

**Authors:** Maarten Vanhaverbeke, Ritienne Attard, Monika Bartekova, Soumaya Ben-Aicha, Timo Brandenburger, David de Gonzalo-Calvo, Costanza Emanueli, Rosienne Farrugia, Johannes Grillari, Matthias Hackl, Barbora Kalocayova, Fabio Martelli, Markus Scholz, Stephanie Bezzina Wettinger, Yvan Devaux

**Affiliations:** Department of Cardiovascular Medicine, University Hospitals Leuven, Herestraat 49, 3000 Leuven, Belgium; Department of Applied Biomedical Science, Faculty of Health Sciences, University of Malta, Msida MSD 2080, Malta; Institute for Heart Research, Centre of Experimental Medicine, Slovak Academy of Sciences, Dúbravská cesta 9, 84104 Bratislava, Slovakia; Faculty of Medicine, Institute of Physiology, Comenius University, Sasinkova 2, 81372 Bratislava, Slovakia; Faculty of Medicine, Imperial College London, ICTEM Building, Du Cane Road, London W12 0NN, UK; Department of Anesthesiology, University Hospital Düsseldorf, Moorenstr. 5, 40225, Düsseldorf, Germany; Translational Research in Respiratory Medicine, IRBLleida, University Hospital Arnau de Vilanova and Santa Maria, Av. Alcalde Rovira Roure 80, 25198, Lleida, Spain; CIBER of Respiratory Diseases (CIBERES), Institute of Health Carlos III, Av. de Monforte de Lemos, 28029, Madrid, Spain; Faculty of Medicine, Imperial College London, ICTEM Building, Du Cane Road, London W12 0NN, UK; Department of Applied Biomedical Science, Faculty of Health Sciences, University of Malta, Msida MSD 2080, Malta; Ludwig Boltzmann Institute for Experimental and Clinical Traumatology, AUVA Research Center, Donaueschingenstraße 13, 1200, Vienna, Austria; Institute of Molecular Biotechnology, BOKU - University of Natural Resources and Life Sciences, Gregor-Mendel-Straße 33, 1180 Vienna, Austria; TAmiRNA GmbH, Leberstrasse 20, 1110 Vienna, Austria; Institute for Heart Research, Centre of Experimental Medicine, Slovak Academy of Sciences, Dúbravská cesta 9, 84104 Bratislava, Slovakia; Molecular Cardiology Laboratory, IRCCS Policlinico San Donato, San Donato Milanese, Milan 20097, Italy; Institute of Medical Informatics, Statistics and Epidemiology, University of Leipzig, Haertelstrasse 16-18, 04107 Leipzig, Germany; Department of Applied Biomedical Science, Faculty of Health Sciences, University of Malta, Msida MSD 2080, Malta; Cardiovascular Research Unit, Department of Population Health, Luxembourg Institute of Health, 1A-B rue Edison, L-1445 Strassen, Luxembourg

**Keywords:** RNAs, Biomarkers, Genomics, Gene expression, Cardiovascular disease, Transcriptomics, Methodology standardization, Translational cardiovascular research

## Abstract

Despite significant advances in the diagnosis and treatment of cardiovascular diseases, recent calls have emphasized the unmet need to improve precision-based approaches in cardiovascular disease. Although some studies provide preliminary evidence of the diagnostic and prognostic potential of circulating coding and non-coding RNAs, the complex RNA biology and lack of standardization have hampered the translation of these markers into clinical practice. In this position paper of the CardioRNA COST action CA17129, we provide recommendations to standardize the RNA development process in order to catalyse efforts to investigate novel RNAs for clinical use. We list the unmet clinical needs in cardiovascular disease, such as the identification of high-risk patients with ischaemic heart disease or heart failure who require more intensive therapies. The advantages and pitfalls of the different sample types, including RNAs from plasma, extracellular vesicles, and whole blood, are discussed in the sample matrix, together with their respective analytical methods. The effect of patient demographics and highly prevalent comorbidities, such as metabolic disorders, on the expression of the candidate RNA is presented and should be reported in biomarker studies. We discuss the statistical and regulatory aspects to translate a candidate RNA from a research use only assay to an *in-vitro* diagnostic test for clinical use. Optimal planning of this development track is required, with input from the researcher, statistician, industry, and regulatory partners.

## Graphical Abstract

**Graphical Abstract:**
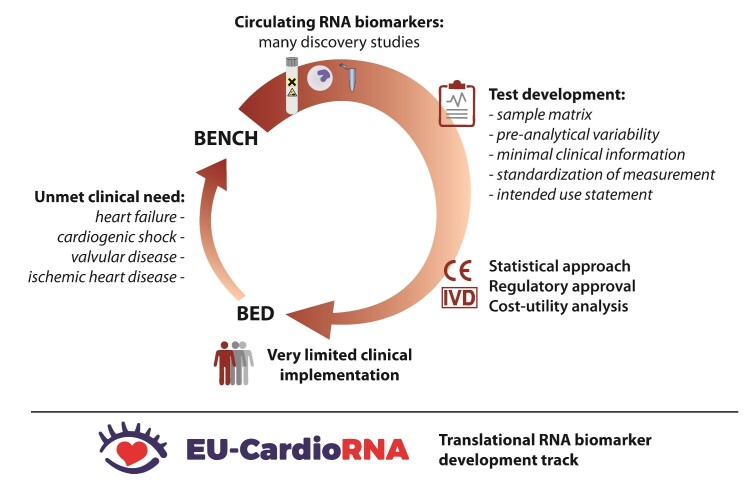

## 1. Introduction

The treatment and outcome of patients with cardiovascular disease (CVD) substantially improved in the past decades. Nevertheless, there is considerable room to improve the diagnosis of CVD, predict disease progression, and tailor therapies accordingly. Recent calls have emphasized this unmet need to improve precision-based approaches in CVD.^1^ Previous studies have shown important pathophysiological roles of coding and non-coding RNAs in all areas of CVD. Since many of these RNAs can be measured in peripheral blood, circulating transcriptome markers are a promising source to molecularly phenotype patients and potentially improve diagnosis, prognostic power, and treatment.^2^ Although a large number of discovery studies have identified candidate RNA biomarkers, only a minority of these markers have progressed towards implementation into clinical practice. The slow development of RNA biomarkers is mainly caused by the complex role of RNAs in disease, discrepant results, difficult and diverse measurement techniques with limited standardization, and lack of samples for large-scale validation.

**Figure 1:**
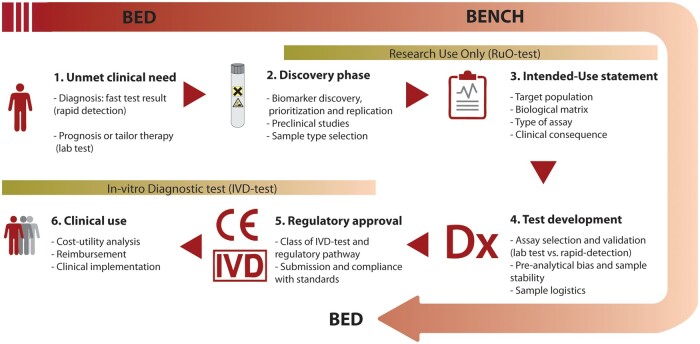
The translational RNA biomarker development track: steps in biomarker discovery, validation, and development of an *in-vitro* diagnostic RNA test from bench to bedside. IVD, *in-vitro* diagnostic; RuO, research use only.

The EU-CardioRNA Action CA17129 is an international consortium supporting collaboration and research on RNAs in CVD, to improve our knowledge of the pathophysiology of RNAs, and to translate this into clinical practice. In this position paper, we describe the translational RNA biomarker development track (*Figure 1*). We systematically list the important steps and pitfalls in the development of RNA biomarkers for CVD. We discuss the unmet clinical needs and address the issues of the different sample types and measurement techniques in the sample matrix. We list potential effect of demographical factors and patient comorbidities, which should be mentioned in each biomarker study. We finally discuss statistical and regulatory issues for translation of the candidate RNA into a clinically validated assay.

## 2. Rationale and clinical need for RNA biomarkers

Coding and non-coding RNAs have important biological functions, which makes them promising biomarkers. Heart- or muscle-enriched microRNAs (miRs) are involved in cardiac development, proliferation, hypertrophy, and failure. For instance, miR-1 and miR-133a are downregulated in cardiac hypertrophy.^3^ Overexpression of these cardiac-enriched miRs was protective against hypertrophy in preclinical models, while inhibition of these miRs resulted in hypertrophy. However, constitutive deletion of miR-133a1 resulted in dilated cardiomyopathy, illustrating the complex biological and temporal mechanism of these molecules. A number of antisense therapies against miRNAs are currently being assessed in phase I clinical studies, e.g. miR-92 and miR-132. miR-132 suppresses genes related to calcium handling (SERCA2A), contractility, and anti-hypertrophic transcription factors (FOXO3). In a preclinical porcine model, monthly intravenous administration of CD132L, an antisense miR-132 inhibitor, improved cardiac function and reversed cardiac remodelling.^4^ In a phase Ib clinical study, CD132L resulted in a dose-dependent miR-132 suppression in patients with heart failure and was well tolerated.^5^

**Figure 2:**
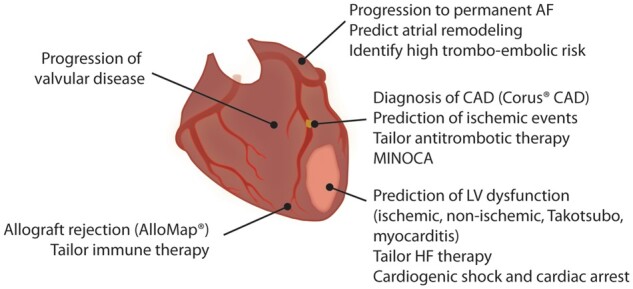
Unmet clinical needs to improve precision-based diagnostics and therapies in cardiovascular disease. AF, atrial fibrillation; CAD, coronary artery disease; HF, heart failure; LV, left ventricular; MINOCA, myocardial infarction with no obstructive coronary artery disease.

Although currently available biomarkers had a significant positive impact on the clinical care of patients with CVD, e.g. high-sensitive assays for cardiac troponins and assays for natriuretic peptides, the prognosis is still poor in many areas of CVD. We next discuss a number of unmet clinical needs where RNAs can potentially improve diagnosis and outcome (*Figure 2*). Because of their important biological function, RNAs may help to identify patients benefitting most from new therapies.

In ischaemic heart disease, muscle- or heart-enriched miRs have been shown to be useful as biomarker to diagnose acute myocardial infarction (MI).^6,7^ However, they currently do not improve the diagnosis of acute MI on top the currently available methods and the turnaround time is long.^8^ Therefore, RNAs in ischaemic heart disease may be more informative to guide therapies after the acute phase, e.g. to improve the prediction of left ventricular (LV) dysfunction post-MI, predict recurrent ischaemic events, and eventually guide new cardioprotective therapies. In patients with ST-elevation MI, adding miR-26b-5p, miR-660-5p, and miR-320a improved the prediction of recurrent ischaemic events.^9^ Thrombo-miRs may also provide new opportunities to tailor antithrombotic therapy in secondary prevention, since tailoring based on platelet function testing did not improve clinical outcome.^10–12^ RNAs may also allow us to better understand the pathophysiology of myocardial infarction with no obstructive coronary artery disease (MINOCA) or type 2 MI, for which no evidence-based therapies are currently available.^13^ Finally, many RNAs related to inflammation and metabolic changes have been identified as promising markers of atherosclerosis and coronary artery disease (CAD).^14^

In heart failure, candidate RNAs such as *QSOX1, LIPCAR*, and *MICRA* have been identified and relate to post-MI LV dysfunction.^15–17^ In contrast to muscle- or heart-enriched miRs, not all differentially expressed RNAs are necessarily released from the heart or mirror changes at the cardiac level.^7,18^ Even in patients with non-ischaemic heart failure, improved molecular phenotyping is required to identify patients who might benefit from upfront intensified neurohormonal blockade or cardiac resynchronization therapy.

In valvular heart disease, the decision to perform a surgical or transcatheter valve intervention is currently made in a multidisciplinary heart team, based on symptoms, echocardiographic parameters, and natriuretic peptides. To optimize the decisions for early intervention, there is a need to improve the identification of patients at high-risk for disease progression, e.g. in aortic stenosis or mitral regurgitation. Both miRs and long non-coding RNAs (e.g. *HOTAIR* and *TUG1*) are dynamically regulated in aortic valve interstitial cells and are associated with inflammation and calcification in patients with aortic stenosis (AS).^19,20^

In atrial fibrillation (AF), besides echocardiographic signs, no biomarkers are available to predict progression of paroxysmal to permanent AF, although treatment options are different. Since the different stages of the disease are accompanied by structural and transcriptomic changes in the left atrium, circulating RNAs (e.g. miR-21-5p and miR-150-5p) may guide us for early detection of progressive disease.^21^ In addition, identifying patients with a high risk of thromboembolism in AF may further improve the risk-benefit ratio of permanent anticoagulation.

Since circulating RNAs act as intercellular communicators, they have the potential to improve the diagnosis of clinical entities involving interactions between the heart and other organs, e.g. cardiorenal syndrome. Similar gaps in the understanding of the pathophysiology of Takotsubo cardiomyopathy, cardiogenic shock, cardiac arrest, and myocarditis lead to poor outcomes, even in the current era.^22^ Very recently, hsa-miR-Chr8:96 has been shown a promising marker for the diagnosis of myocarditis, discriminating patients with myocarditis from healthy subjects and patients with MI.^23^ The marker was validated in a preclinical model and in three moderately sized independent cohorts. However, as for almost all of the aforementioned examples, it remains unknown to what extent these markers may improve treatment decisions and improve outcome.

## 3. Sample collection and processing

**Figure 3:**
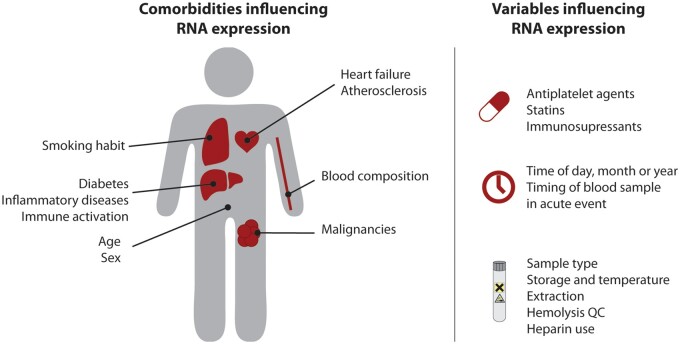
Comorbidities and preanalytical variables with significant impact on RNA expression. QC, quality control.

Sample collection is a crucial step when using RNA-based biomarkers. Different sample types are available to collect cell-free or cellular RNA (the sample matrix, *Table 1*). Although RNAs from cellular subfractions or extracellular vesicles (EVs) yield more specific RNA expression patterns, they are more time consuming to process and to store compared to plasma or whole blood. Many factors in sample collection and processing can result in increased preanalytical variation with subsequent deleterious impact on the quantification of the biological variability. These preanalytical variables should be reported in each biomarker study (*Figure 3*).

**Table 1:** Advantages, pitfalls, and recommendations for each sample type in sample preparation and RNA measurement (sample matrix)

Sample collection and processing		
	Advantages (+) and pitfalls (–)	Recommendations

Serum or plasma	+ Easy to obtain and to store + Often available from biobanks − Cellular contamination − Storage temperature and platelet activation − Interference by haemolysis	EDTA or CTAD (to prevent unintentional platelet-derived RNA contamination) as preferred anticoagulant. Avoid heparin Use miR-16, miR-451, or miR-486 for haemolysis quality control Assess and standardize sampling temperature
EVs	+ Potentially cell- or disease-specific information − Time-consuming isolation (ultracentrifugation) − Unknown purity when using commercially available kits	Verify purity when using commercially available kits Consider prefiltering sample, excluding particles larger than 0.8 µm
Whole blood or isolated cells	+ Easy to obtain and to store (whole blood) − Lower sensitivity or specificity to detect a meaningful expression profile because assessing bulk RNA (whole blood) − Globin decreases available reads in RNA seq (whole blood) − Impact of differential cell counts (whole blood) − Time-consuming isolation (isolated cells)	Commercially available tubes with RNA stabilization agent (whole blood, e.g. PAXgene^®^ blood RNA system, PreAnalytiX, Hombrechtikon, Switzerland) Library-prep with globin depletion (e.g. Illumina Stranded Total RNA Prep with Ribo-Zero Plus) Report or normalize for leucocyte subset counts

**RNA extraction and measurement**		

	Advantages (+) and pitfalls (–)	Recommendations

Serum or plasma	− Low yield, difficult RNA quantification − Phenol-based extraction: higher yield, lower purity and inhibition of downstream reactions. − Column-based extraction: each kit with different yield and size cut-off − RNA inhibition and spurious expression levels caused by heparin − Endogenous controls difficult to identify − Risk of sequencing bias during small RNA sequencing	Phenol-based: if low yield add carrier. If low purity, consider ethanol precipitation or column-based clean-up (e.g. Qiagen RNeasy^®^ MinElute^®^ Cleanup). Be careful to retain small RNAs. Consider heparinase treatment RNA quantification: standardize initial sample volume and monitor PCR efficiency Use a combination of endogenous controls and exogenous spike-ins (e.g. cel-miR-39) for normalization. Specific protocols to prevent sequencing bias (e.g. circularization, unique molecular identifiers)
EVs	− Very low RNA yield with difficult RNA quantification − Endogenous controls difficult to identify	Similar to serum/plasma Lyse EVs before RNA isolation Prefer no preamplification and consider digital PCR Column-based RNA extraction has acceptable yield
Whole blood or isolated cells	+ High RNA yield + RNA purity and yield easily tested (e.g. spectrophotometric) + Endogenous controls can be used	Identify and validate proper endogenous control (e.g. using the NormFinder or GeNorm algorithm)

CTAD, citrate–theophylline–adenosine–dipyridamole; EDTA, ethylenediaminetetraacetic acid; EVs; extracellular vesicles; ISEV, international society for extracellular vesicles; PCR, polymerase chain reaction.

For the collection of cell-free RNA, the use of serum tubes or tubes with anticoagulants [ethylenediaminetetraacetic acid (EDTA), sodium citrate, or heparin] differentially affects the concentration of miRs. The repertoire and concentrations of RNAs found in serum samples differs to those of corresponding plasma samples due to RNAs released during the coagulation process.^24^ Significant variation in cell-free RNA plasma levels can occur due to platelet contamination since platelets are specifically rich in miRs (e.g. miR-24, miR-126, miR-191, miR-223).^12^ The use of citrate–theophylline–adenosine–dipyridamole as anticoagulant has been suggested to decrease contamination related to platelet activation.^25^ Platelet-poor plasma can also be used to decrease contamination by platelets and their RNA content. Heparin as an anticoagulant should be avoided since it has a dose-dependent inhibitory effect on enzyme-based RNA quantification.^26^ The same recommendation should be taken into account for samples from patients treated with heparin as anticoagulant.^27^ The use of heparinase to remove heparin from the samples may be considered.^7^ Finally, since blood cells are a significant source of RNAs, perturbations in blood cell counts and haemolysis may alter plasma miR levels by up to 50-fold.^18^ Different methods have been developed to screen serum and plasma samples for haemolysis, e.g. including miR-16, miR-451, and miR-486, as quality control downstream the RNA quantification process.^28^ To reduce haemolysis caused by blood sampling, a needle of adequate gauge should be used.

The storage conditions of the blood samples as used in clinical routine may affect the extracellular RNA profile before the isolation of cell-free fractions. Although some families of RNAs, such as miRs and circular RNAs, have been reported as highly stable in clinical specimens due to their remarkable resistance to nucleases, both miR degradation and release of RNA from cellular components influence the cell-free RNA profile.^29,30^ Köberle *et al.*^31^ found that levels of miR-1, miR-16, and miR-21 were increased in EDTA and serum collection tubes incubated for 1–3 h at room temperature prior to separation of plasma or serum from cell components, even in the absence of significant haemolysis. Differences in temperature and coagulation times may increase the variability between samples. As such, delayed processing of samples and inconsistency in storage temperature may introduce significant technical variation. A rapid specimen processing, i.e. within 1–4 h as recommended by some standard operating procedures, and consistency in storage temperature is recommended.^32^ Nonetheless, this is not always possible, e.g. in multi-centre setting. A meticulous storage documentation and detailed quality control experiments are therefore imperative. The use of RNA stabilization reagents may also be considered.^33^ Finally, the contamination of blood with epithelial cells during puncture can be avoided by discarding the first 1–2 mL of blood drawn.^34^

Besides cell-free RNA fractions, whole blood is also an excellent source of RNA-based biomarkers. The transcriptomic signatures of blood cells could provide valuable information on disease status and progression. The development of commercially available blood collection systems allowing RNA stabilization and facilitating the storage of the samples makes whole blood very convenient for clinical practice. Subtle or cell-specific changes in RNA expression may not be detected since the bulk RNA profile is measured of the entire blood compartment. Depletion of abundant red blood cell-derived RNAs (e.g. globin RNA, miR-16, or miR-486) may be required to improve the sensitivity of micro-array or RNA sequencing studies.^35^ RNA profiles obtained from isolated cells, e.g. CD14+ monocytes or specific lymphocytes subsets, using fluorescence-activated cell sorting (FACS) or magnetic bead isolation, may be more specific but they are time consuming to collect and process.^36^

## 4. Extracellular vesicles

In addition to the aforementioned sample types, EVs are a promising source of circulating biomarkers with specific characteristics. EVs exist in different sizes and are released by the parent cells into the circulation, shuttling molecular information, including miRNAs, from the parent cells to the extracellular space.^37^ EVs therefore have a good potential to translate into minimally invasive biomarkers (liquid biopsy).

Larger EVs, such as microparticles, possess a panel of surface markers, which allow tracking of their cellular origin, particularly from platelets, endothelial cells, leucocytes, and erythrocytes. Despite some initial attempts, protocols enabling tracking of the cellular origin of the smaller EVs populating the biofluids of patients are still lacking. This could improve with recently introduced platforms, such as the ExoView R100 (NanoView Biosciences, Brighton, US) and the Flow Nanoanalyzer (NanoFCM, Nottingham, UK). The former is an automated platform, providing multi-level measurements for exosomes and other small EV particle size and concentration analyses, and membrane antigens colocalization. The latter reportedly acts as a high sensitivity flow cytometer combining light scattering and fluorescence detection with distributions of particle size. These techniques could potentially support the use of EVs as circulating biomarkers without the need to purify them. While researchers have mostly focused on the internal cargo of EVs, potential biomarkers are indeed also present at the level of the EV external surface.

To study the molecular content of EVs, the best approach is still to enrich these vesicles from the biofluid before performing molecular analyses. The comprehensive analysis of several studies on pools of EVs extracted from a biofluid suggests EVs could contain proteins, microRNAs, other RNAs, and DNAs as well as metabolites, such as sugars, amino acids, lipids, nucleotides, and *N*-glycans.^37^ The molecular signature of individual EVs is still debated, with some papers suggesting not more than one miRNA present per every 1–100 EVs.^38^ Definitive conclusions are understandably difficult, given the current infeasibility of profiling the molecular content at single EV level, and when considering that different protocols for EV isolation and RNA or protein extraction and analysis produce different profiles from the same biofluids.^39,40^ These discrepancies will be probably resolved as technologies advances.

Recommendations on minimal information that needs to be reported when performing studies with EVs have been published by International Society for Extracellular Vesicles.^41^ These include storage temperatures of the source (e.g. plasma), the volume used for EV extraction, the extraction method with the trade-off between recovery, and specificity and quantification by at least two methods. Recommendations on EV isolation and RNA extraction are summarized in *Table 1*. The most used isolation methods remain density gradient centrifugation, ultracentrifugation, and their combination with size exclusion chromatography. However, these techniques are time consuming, require specialized equipment, and are difficult to automate, which poses a limitation for the use in clinical practice. The effect of using more simplified, cost-effective, and commercially available methods (often resulting in higher recovery but lower EV specificity) needs to be assessed when developing an EV-based biomarker. Contamination caused by the method of EV extraction also needs to be considered, especially for preparation of serum or plasma EVs, which usually carry lipoproteins.

Despite these technical limitations, work on pooled circulating EVs clearly show disease-associated molecular changes and suggest the potential of EVs as diagnostic and prognostic biomarkers. For instance, the number of monocyte-derived microparticles related with outcome in patients with ST-segment MI, while the specific cargo in monocyte-derived EVs (e.g. decreased *MTCOI*) related to poor outcome in patients with stable coronary disease.^36,42^ In contrast to the oncological field, clinical studies with EVs in CVD are still in the discovery or recruitment phase (NCT04349189, NCT04191044, NCT04327635, NCT01104220).

## 5. RNA measurement

After having assessed sample collection and the appropriate sample type, RNA extraction poses challenges that vary according to the sample type, analytical method, and future automation requirements (*Table 1*). RNAs are generally extracted using either a traditional phenol-based nucleotide extraction method (e.g. TRIzol) or silica column-based extraction kits. In this latter case, the RNA species being tested (small, e.g. miRs, or long, e.g. mRNAs) guides the choice of the kit with the appropriate size cut-off. Among plasma extracellular miRNA isolation kits, the miRNeasy kit from Qiagen (Hilden, Germany) performs well in terms of purity and recovery, but results in a high amount of unmapped reads in RNA-sequencing.^43^ The low RNA yields derived from plasma, serum, and EVs are a significant limiting factor. Adding carriers, such as glycogen, may also improve yield in RNA-precipitation-based protocols. Due to the membranous nature of the exosomes, lysis should be performed before RNA isolation from EVs following the manufacturer’s protocol. A pure RNA extract for downstream analysis is still preferred above preamplification, since the latter step may increase variability and bias. It is important to be consistent when performing RNA extraction and measurement: only when using standardized protocols, the results can be interpreted and compared correctly.^44^ For whole blood samples, the inference of haemoglobin should be considered, which, along with heparin, is one of the major sources of inhibitors for downstream RNA amplification. RNA quantification and quality checks are generally performed using spectrophotometric (e.g. 260 and 260/280 nm absorbances) and electrophoresis (e.g. Agilent 2100 Bioanalyzer) techniques. However, this step is generally not possible for RNAs derived from plasma or serum, due to the low amounts recovered. A commonly used strategy consists in extracting the same volume of plasma or serum, and monitor the efficiency of the extraction and measurement steps [e.g. reverse transcription and polymerase chain reaction (PCR)] using synthetic spike-ins (e.g. cel-miR-39, Quanto EC1 and 2).^45^ The detection of synthetic spike-ins seems to be particularly sensitive to heparin inhibition.

RNA measurement techniques can be divided between high-throughput and targeted approaches. Among the first, RNA-sequencing and microarrays allow to investigate the whole transcriptome or a large portion of the sample, respectively, providing a broad analysis that is particularly useful during the biomarker discovery phase.^46^ Specifically, high-depth RNA-sequencing, long-read isoform sequencing, and exon-arrays are not only quantitative and sensitive, but can also provide information on the structure of the transcripts analysed.^47^ Single-cell RNA sequencing can be used to identify transcripts in rare circulating cell types, e.g. obtained using FACS. High-throughput techniques, however, are relatively time consuming, expensive, require specialized instruments, skilled technical personnel, and a complex bioinformatics analysis. Plasma/serum miRNA profiling, in particular, poses a variety of challenges that may significantly affect the results, stimulating the development of specific strategies to circumvent them: the formation of adapter dimers may be prevented using chemical modifications or a single-adapter and circularization approach.^48,49^ The latter protocol has also been shown to reduce the ligation bias that can negatively affect the coverage of low abundant miRNAs. Moreover, introducing unique molecular identifiers in the adapter sequences can attenuate the bias ensuing from PCR duplicates.^50^ Considering all these potentially confounding elements, validation of profiling results with an independent technique [e.g. quantitative PCR (qPCR)] is strongly advised.

Among the targeted measurement techniques, qPCR represents the gold standard. This amplification-based assay is an accurate method to measure RNA and sufficiently sensitive to be reliably used in plasma and serum samples. It is also cost-effective and not particularly challenging from a technical and data-analysis point of view. For the detection and accurate quantification of low-abundance RNAs (e.g. plasma, serum, EVs), digital PCR (dPCR) is particularly indicated, as it provides direct, absolute, and precise measures of target sequences, although in a less cost-efficient manner compared to qPCR.

The next crucial issue is the choice of the correct reference RNAs for expression level normalization. This is particularly challenging for plasma/serum miR studies, where exogenous spike-in RNAs are still frequently used because of the lack of widely accepted endogenous normalizers.^51^ While exogenous spike-ins are very useful in monitoring the efficiency of extraction and measurement, they do not account for the quality of the original sample. Endogenous normalizers are, theoretically, the best choice, and should have low deviation of expression levels across samples, be minimally affected by storage conditions and sample processing, and display a maximal efficiency of extraction.^45^ A commonly used normalizer is miR-16. However, this miRNA is present in significant levels in red blood cells and its levels in the plasma are proportional to the degree of haemolysis. Thus, its value as normalizer is limited to sample sets in which haemolysis is tightly controlled. Small non-coding RNAs RNU6A and RNU6B are among the reference genes most frequently used as normalizers. Although they have many positive characteristics, they are not miRs and their normalization value can always be questioned.^45^ When an RNA expression panel is available from a profiling study, the total RNA content of the sample can be used for global normalization or the panel can be used to identify the most suitable endogenous normalizer for the specific experimental setting. In order to compensate the potential shortcomings of each individual normalization method, a combination of endogenous and exogenous controls is recommended.

Each of the above-mentioned RNA detection techniques currently requires considerable time and advanced expertise for sample preparation and analysis, making them adequate platforms for centralized laboratories, but not suitable for point-of-care diagnostics. Alternative technologies hold great promise, such as isothermal amplification (e.g. loop-mediated isothermal amplification), nanobead-based, oligonucleotide-templated reaction-based, electrochemical signalling-based, lateral flow assay-based, and microfluidic chip-based strategies.

## 6. Influence of patient characteristics and comorbidities on RNA expression

Cardiovascular risk factors and comorbidities (e.g. diabetes and obesity) act, at least in part, through alterations of metabolic and inflammatory profiles. A growing body of literature also suggests that these factors, together with demographic factors, influence levels of peripheral blood RNAs, highlighting the importance of documenting, reporting, and if necessary adjusting for these confounding variables in biomarker studies (*Figure 3*). We will here discuss those variables with sufficient evidence to at least partially affect RNA expression in cellular or cell-free fractions (*Table 2*).

**Table 2:** Comorbidities and covariables in cardiovascular patients that impact peripheral blood RNA expression

Category	Description
Sampling and timing	*Circadian rhythms*. Diurnal (cell-free RNA)^52^ and seasonal (cellular RNA) changes.^53^ Changes in plasma miRs after exercise^54–56^ *Storage and processing*. Changes depending on storage time, sample processing^57^ and heparin use^7^
Demographics	*Age*. Impact on both cell-free^57,58^ and cellular^59,60^ RNAs *Sex*. Variation in cellular RNAs according to sex^61,62^ and menstrual cycle (cell-free RNA)^63^
Risk factors	*Smoking habit*. Impact on cellular RNAs,^64,65^ sometimes reversible, and cell-free RNAs. *Diabetes*. Cellular RNA significantly influenced by diabetic status in different cohorts (using both micro-array as well as qPCR).^14,62,66^ *Obesity and metabolic syndrome*. Changes in cellular RNA.^66,67^*Hypertension* (cellular)^68^
Non-cardiac comorbidities	*Inflammatory disease*. Differences observed in asthma,^69^ COPD (cellular RNA),^70^ systemic lupus erythematosus,^71^ and ankylosing spondyloarthritis^72^ *Oncological disease*. Numerous circulating coding and non-coding RNAs have been identified in cellular and cell-free fractions of patients with malignancies^73^ *Immunologic events*: Changes in patients with kidney transplant rejection^74^
Cardiac comorbidities	*Atrial fibrillation*. Differential expression in cellular and plasma fractions of patients with prevalent atrial fibrillation vs. controls^21,75^ *Heart failure*. Cell-free RNAs in systolic (e.g. *LIPCAR*)^16^ and diastolic (*SENCR* and other miRs)^76,77^ heart failure. Cellular RNAs in ischaemic heart disease (e.g. *QSOX1, MICRA*)^15^ *Atherosclerosis*. Many differentially expressed RNAs have been described in cell-free fractions,^6,78^ monocytes,^36,67,79^ and whole blood^62^ *Valvular disease*. Possible changes in plasma miRs depending on aortic stenosis severity, with interactions based on the presence of coronary atherosclerosis^20,80^
Concomitant cardiovascular drugs	*Statins*. Differential RNA expression in whole blood in a COPD cohort^81^ *Antiplatelet drugs*. Platelet miR changes in patients treated with aspirin and clopidogrel^82^ Changes in cell-free RNAs in patients treated with aspirin^83^ *Immunosuppressants*. High doses of corticosteroids (AlloMap^®^ in the CARGO study using PBMCs^84^)
Acute events	Significant changes in many different cohorts of patients with *acute MI* (both cellular and cell-free),^6,15^*stroke* (cellular),^85^ and *acute infection*^86^

COPD, chronic obstructive pulmonary disease; MI, myocardial infarction; miRs, micro-RNAs; PBMCs, peripheral blood mononuclear cells; qPCR, quantitative polymerase chain reaction.

Age is one of the factors most strongly associated with changes in both miR and mRNA expression, driven by cellular senescence and age-related metabolic conditions.^57–60^ Lifestyle factors such as alcohol consumption and smoking also effect expression of mRNA and miRs. Changes due to smoking have been observed in multiple tissues including airway epithelial cells, lymphocytes, and peripheral whole blood.^64,65^ Many of the genes influenced by smoking are those involved in the modulation of the immune system, blood coagulation, and natural killer cell and cancer pathways.^58,65^ The differential gene expression associated with smoking may be due to its impact on chromatin remodelling and DNA methylation status, related to peroxidation products and direct DNA damage by smoking components. Some of these effects are reversible while others persist after smoking cessation.^58^ Alcohol has also been shown to induce DNA methylation changes that effect transcription.^87^

The effect of fasting on miR expression profiles in humans remains unclear.^88^ In preclinical models, fasting upregulated miRNA-induced silencing complex components, which in turn regulates a wide range of miRNAs.^89^ There is ample evidence that diet influences miR expression, whilst there is conflicting evidence about food-derived miR ending up in the circulation.^90^ Despite a high degree of interindividual variation in gene expression, a recent study on whole blood transcriptomes highlighted effects due to fasting and post-prandial status after a high-fat meal, with some genes, including circadian rhythm genes, exhibiting a universal response, while others, including innate immune response genes, exhibiting subject- and time-dependent differences in response.^91^ Dietary patterns have also been associated with sex-specific differential expression of thousands of mRNA transcripts in peripheral blood mononuclear cells.^92^ Physical exercise has also been shown to influence mRNA and circulating miR levels in both acute and chronic scenarios.^54–56^

External factors such as seasonal changes have been shown to influence both miR and mRNA profiles mostly through widespread seasonal changes of immune cells and their gene expression.^53,93,94^ Circadian rhythms may cause variations in circulating miR levels, thus timing of blood collection (day/night) might affect miR expression. Several rhythmically expressed miRs have been documented in various tissues including heart and blood plasma in animal studies.^95^ Robust diurnal oscillations have also been found for tissue Dicer expression (endonuclease controlling miR processing) in mice.^96^ Only a limited number of studies focused on the diurnal variations in humans. While in few studies, no circadian variations in levels of circulating RNAs were found, Heegaard *et al.* found clear rhythmicity in circulating levels of 26 miRs showing out two main diurnal phase patterns.^52,96,97^

Hormonally-controlled cycles, primarily menstrual cycle in women, represent additional physiological rhythmicity possibly influencing miRs expression. Bovine plasma levels of selected miRs distinctly increased during oestrus as compared to other stages of the cycle, while data on miR expression during the hormonal cycle in humans is inconclusive so far.^98^ While Rekker *et al.* and Max *et al.* found no changes in circulating miRs levels within the menstrual cycle in healthy women, Eisenberg *et al.* found elevated serum levels of selected miRs in early follicular phase as compared to early luteal phase of the cycle.^63,88,99^ More generally, sex is considered to significantly affect expression levels.^61,62^

Cardio-metabolic risk factors, which include body mass index, hyperlipidaemia, diabetic status, and hypertension, have been shown to influence both mRNA and miR levels in research subjects from the Framingham Heart Study selected to exclude those on medication for hypertension, dyslipidaemia, or diabetes.^100^ Whole blood gene expression data identified specific mRNA transcripts that were upregulated amongst obese individuals, most of which belong to pathways associated with the metabolic state and inflammatory responses.^101^ Many studies have identified changes in circulating RNA expression in patients with diabetes mellitus, with significant overlap with obesity, metabolic syndrome, and CAD.^14,62,66,67,102^ Whole blood mRNA expression signatures were also associated with hypertension.^68^ The effect of LDL cholesterol levels on the expression profile were linked to oxidized low-density lipoprotein (ox-LDL), with ox-LDL-loaded cells showing transcriptional changes in expression of pro-inflammatory genes.^103^ The evidence for other cardiovascular and non-cardiovascular comorbidities and drug therapies is further summarized in *Table 2*.

## 7. Statistical approach for biomarker discovery and validation

**Figure 4:**
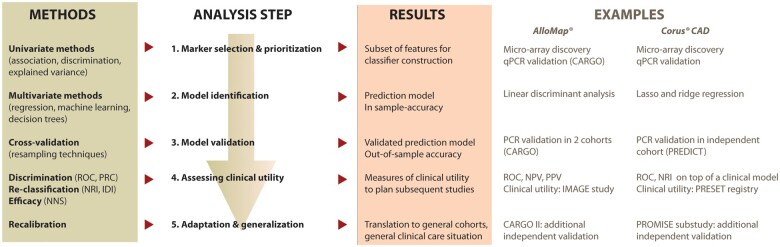
Statistical workflow to develop and validate an RNA-based classifier and assess the clinical utility. CAD, coronary artery disease; IDI, integrated discrimination index; NNS, number needed to screen; NRI, net reclassification improvement; PRC, precision-recall curve; qPCR, quantitative polymerase chain reaction; ROC, receiver operating characteristics.

State-of-the-art detection and validation of biomarkers and related scoring systems relies on thorough data analyses comprising multiple steps. Although there is no common, generally accepted standard of this process, most biomarker research papers follow a general approach (*Figure 4*). For each of the steps, there is a plethora of available statistical methods and there is ongoing discussion, which performs best in which situation.

### 7.1 Step 1: marker selection and prioritization

In the first step, features for RNA biomarkers are selected from data of high-throughput technologies such as RNA sequencing. After proper technique- and study-specific pre-processing and quality control, we have a situation of a large number of markers (M) available in a small number of individuals (N, N often much smaller than M). Therefore, in the first step of biomarker discovery, dimensionality of the problem is often reduced by selecting or prioritizing markers for the subsequent analysis steps. This step is not mandatory and could be combined with the model identification step (see below). Methods to deal with this problem are from the class of (univariate) variable importance analyses and comprise for example methods of univariate hypotheses testing, explained variance analysis, regression techniques, or methods of discrimination analysis.^104^ If required, correlation structure of features can also be considered during this step, e.g. by removing correlated features or applying de-correlation techniques.^105^

### 7.2 Step 2: model identification

In the next step, markers selected at Step 1 are combined to a prediction model, i.e. a formula or decision tree combining the information of several features to one predictive framework. Established biomarkers or clinical covariates can also be included. This step typically includes model selection and parameter estimation for which several methods and concepts are available comprising for example multivariable regression and classification approaches, which could be linear (e.g. logistic regression, linear discriminant analyses, and support vector machines), non-linear (e.g. support vector machines with non-linear kernels, k-nearest neighbour with different distance measures), or based on decision trees or graphical models. Advantages and disadvantages of these methods are compared elsewhere.^106^

Overfitting is a serious concern during establishing a classifier, i.e. the classifier typically performs better in the data set where it was developed (training set) than in a new data set (validation set). Several measures are applied to reduce this issue as much as possible comprising for example penalization approaches (information criteria, shrinkage) or prior limitation of the degrees of freedom of a model.^107^ The goal is to find a trade-off between the accuracy of the prediction in the training set (also called in-sample accuracy) and a new data set (out-of-sample accuracy).

### 7.3 Step 3: model validation

Estimation of the out-of-sample accuracy requires an independent data set comparable to that of the training set. If such a data set is not available, the set of samples can be split into a training set and a validation set, i.e. a subset of samples is not used for the analyses in Step 2 but reserved for assessing the prediction accuracy of the model in Step 3. To avoid dependence of model identification and validation on a specific split of the data, methods of cross-validation are often applied.^108^ This means that the splitting of data is repeated in a systematic manner and a consensus model is constructed. Combining cross-validation with resampling techniques for improved out-of-sample prediction is proposed.^109,110^

### 7.4 Step 4: assessing clinical utility

Assessing the potential clinical impact of a new classifier is an important step towards bedside translation. Several aspects are of interest. First, the discriminative power of the test needs to be evaluated, e.g. by receiver operating characteristics (ROC) allowing assessing sensitivity and specificity of the test for a given cut-off. Often, defining a cut-off is not straightforward due to uncertainty with respect to the costs of false negatives and positives. Therefore, the area under the curve (AUC) is typically considered as a measure of discriminative strength. A complementary characteristic are precision-recall curves displaying the relation between the positive predictive value (precision) and the sensitivity (recall). Compared to ROC, this curve is not affected by imbalanced case/control samplings, e.g. enrichment of controls with low classifier values.

In the next step, new classifiers are compared with current standards (other biomarkers, tests, risk factors). Although this can be done by comparing, e.g. AUC of ROC, there are clinically more intuitive indices available for this purpose. Net Reclassification Improvement (NRI) compares the correct re-classifications between two tests.^111^ It is recommended to calculate this statistic separately for cases and controls.^112^ The Integrated Discrimination Index (IDI) assesses the gain in sensitivity and specificity over all possible cut-offs compared to another test.^113^ It was pointed out recently that NRI and IDI should not be used for significance testing.^114^ Finally, the effectiveness of a new classifier in a given population can be evaluated by Numbers Needed to Screen, which is the average number of tests required to prevent one event.^115^ However, this statistic also depends on the effectiveness of treatment measures applied to test-positives.

### 7.5 Step 5: adaptation and generalization

Generalization is a major issue to improve clinical utility of a developed classifier. Often, the classifier was developed in a specific selection of cases and controls (i.e. with specific inclusion/exclusion criteria and highly standardized testing environments), which does not mirror the general population or conditions in general health care. Thus, the classifier might require re-calibration to new situations by re-weighting the components of the prediction model, e.g. by using appropriate regression techniques to determine new weights or by Bayesian approaches using the established weights as priors.

## 8. Regulatory aspects from research use only to *in-vitro* diagnostic test

Once potential biomarkers have been evaluated in a discovery phase, steps have to be made towards the development of a clinically useful assay. Only few candidate markers from a discovery phase are translated to the status of research use only (RoU) tests. Even more rare is that RoU tests are validated as *in-vitro* diagnostic (IVD) tests and taken up into clinical practice. Thus, we observe that only few promising projects are continued beyond the publication of interesting biomarker discovery and validation studies, or even the RoU status of development. Besides the need for reproducibility in broader validation cohorts, there are other important milestones that need to be achieved in order to translate these biomarker candidates into clinically useful IVD tests. We next describe these other crucial steps that need to be taken to go from biomarker validation to approval as an IVD test (*Figure 1*). Some of these steps may already have been partially addressed during the discovery or validation phase of the study.

### 8.1 Step 1: the ‘Intended-Use Statement’

The first essential step, if not done already at the beginning of the discovery phase, is to define the ‘intended-use’ of the test. This seems a trivial action but is highly important as it will subsequently affect the regulatory pathway and efforts that need to be taken during analytical and clinical validation of the biomarker. The intended-use must include a description of the (patient) target population, the biological matrix, the type of assay (quantitative vs. qualitative), and target molecules, and potentially the clinical action that is triggered by the test. From our experience, the clinical uptake of a test strongly depends on the clear definition how the test result will support decision-making of physicians.

### 8.2 Step 2: assay selection, validation, and sample logistics

In the next step, the requirements for assay type and sample logistics can be delineated from the intended use. Typically, the assay platform for biomarker discovery and validation is selected based on the availability of instruments and know-how in the research lab, and commonly RNA biomarkers are measured using various types of laboratory assays such as qPCR, dPCR, RNA sequencing, or other targeted hybridization-based techniques. However, the turn-around-time that is required for the test result to be clinically useful must be considered as well. In case the turn-around-time can be 6–8 h or more, conventional ‘laboratory assays’ such as qPCR are suitable. In this case, the pre-analytical logistics such incubation times before sample processing, and storage temperatures and analyte stability must be determined alongside analytical validation of the assay.^116^

In case the turn-around-time should be shorter, rapid diagnostic test platforms or point-of-care platforms must be used for the IVD test.^117^ This will likely require additional assay development efforts and bridging studies to demonstrate comparability to the results obtained from the laboratory tests. Once the assay and sample type have been determined, manufacturing protocols according to for example ISO13485 standards must be implemented. The prototype assays need to be taken through analytical validation studies to determine key parameters such as reproducibility, accuracy, specificity, and sensitivity of the test. Analytical validation protocols should be defined taking into account the national or regional legal requirements for IVD approval.

### 8.3 Step 3: regulatory pathway

In Europe, the legal basis for placing IVD tests on the market is the *In-Vitro* Diagnostics Regulation (IVDR), which was released in April 2017 and will fully replace the *In-Vitro* Diagnostics Directive after 26 May 2022. Importantly, the IVDR includes a risk-based classification of IVDs with four risk classes (A is lowest risk, and D is highest risk), which take into account the intended purpose of a test as well as its inherent risk. For example, tests for determining blood type to determine the suitability of blood specimens for transfusion are considered high risk, since a wrong test result will have severe impact on the health of the test person. A major change compared to the previous directive is the risk classification system, since the IVDR risk classification is now based on rules, which means that the intended-use of the test together with assay type define the risk class. For example, most ‘omics-based’ technologies for diagnosis, prognosis, staging, or guiding the treatment of a disease will from now on fall into the second highest class C, which means mandatory involvement of notified bodies (which are tasked with IVD approval in Europe) and conduction of clinical performance evaluation studies using the analytically validated test device. The extent of such studies depends on the intended-use (i.e. the type and size of the disease population) and the already available amount of data.

In the United States, the FDA is tasked with the approval of molecular diagnostic IVD tests and distinguishes three risk classes. Molecular diagnostic tests without predicate devices, i.e. already approved tests with the same intended-use and technology, will most likely require clinical studies. In order to determine the exact regulatory pathway [510(k) pre-market notification vs. pre-market approval] a pre-submission meeting with the FDA is highly recommended.

### 8.4 Step 4: cost-utility analysis and reimbursement

Once the technical documentation of the analytical and clinical performance of an IVD test have been reviewed and approved by the regulatory agencies, IVD tests can be placed on the market. However, this does not necessarily result in immediate uptake of the test by its target audience. Uptake of a novel test can be catalysed by two main achievements.^118^ First, inclusion into patient management and treatment guidelines by any professional society, and second reimbursement of the test cost by medical insurances. In order to achieve this, the impact of any new IVD test on health of the target population and the budget of the paying stakeholder needs to be evaluated. This can be done using the principals of cost-utility (or health-economic) studies, which model the impact of test uptake on health outcomes such as mortality, morbidity, life-years, and quality-adjusted life years.^119^ The change (improvement) in health outcomes is then contrasted with the expected impact on budget for the IVD test, additional (or avoided) treatment, and/or hospital interventions, etc. Cost-utility is then calculated as the incremental cost-efficiency ratio between the change in health (H) and change in budget (B) before and after uptake of the IVD test: $\mathrm{ICER}\;(\frac{€}{\mathrm{health}})=\;\frac{H_{1}-H_{0}}{B_{1}-B_{0}}$. The amount of money paid per unit of health outcome (‘willingness to pay’) is country-specific, but as a rule-of-thumb, one gross domestic product per unit of health outcome is considered cost-effective, meaning that health care payers are willing to invest this money. Importantly, the construction of such a model can be very helpful during the early stage of IVD test development since it can be very helpful to define thresholds for diagnostic performance and cost of the test procedure in order to be still ‘clinically useful’.

## 9. Examples of clinical-grade RNA-based tests

To our knowledge, only two clinically relevant RNA-based IVD tests have been made commercially available: AlloMap^®^ (CareDx, Brisbane, CA, USA) to rule-out acute cellular rejection after cardiac transplantation and Corus^®^ CAD (CardioDx, Palo Alto, CA, USA) to rule-out CAD in non-diabetic patients. Both tests were developed using an RNA micro-array in the discovery phase, followed by qPCR to confirm the identified genes (on the same or new samples) and to establish the classifier (*Figure 4*).^14,84,120^ Importantly, preanalytical bias caused by clinical variables affecting expression of the transcripts was timely identified and corrected for (e.g. time post-transplant, steroid dose, and transcripts influenced by sample processing for AlloMap^®^ and diabetic status and cell count for Corus^®^ CAD). The classifier was next validated in independent cohorts.^78^ The clinical benefit of AlloMap^®^ was shown in the IMAGE study, with a reduction in the number of endomyocardial biopsies without an increase in adverse events.^121^ This clinical benefit, together with continued validation (CARGO II) and regulatory approval, led to active clinical use of this RNA-based test.^122^ For Corus^®^ CAD, additional validation studies were performed, but the clinical benefit was less convincing and no regulatory approval was obtained.^123,124^ Together with the availability of other non-invasive tests to rule-out CAD (such as coronary CT angiography), this led to the discontinuation of the test.

## 10. Perspectives and main challenges

There is a clear need for new molecular biomarkers for clinical use. However, only a well-orchestrated approach using standardized techniques and showing clinical benefit can lead to a successful IVD test. With COVID-19, we have seen an unprecedented interest in the use of RNA-based vaccines. These developments will certainly pave the way for future developments of other RNA-based applications.

The potential of RNAs to aid in personalizing health care has been suggested by many studies, yet RNA-based biomarker applications are still at a preclinical research level. Challenges linked to the measurement of RNAs in biological fluids are currently being addressed and great efforts are being made regarding their reproducibility, accuracy, time- and cost-effectiveness, and acceptance by the clinical community. Because of the complex RNA biology, the exact mechanism which results in a dysregulated peripheral blood level of a candidate RNA biomarker is often uncertain, e.g. as for *LIPCAR* or *QSOX1*, while for others, e.g. muscle-enriched miRs, it is clearer. Therefore, to facilitate uptake by the clinical community and approval by the regulatory bodies, independent validation of associations between RNA expression values and disease outcome in properly sized populations is of paramount importance. A database gathering existing cohorts of patients with CVD available for sharing will help designing independent validation strategies.^125^ Since some RNAs have a significant variability in expression levels, prediction models based on artificial intelligence and combining both RNA data and clinical data of patients may constitute future tools for personalized medicine.^126^

With these recommendations, we aim to promote standardization in the development process and catalyse efforts to investigate novel RNAs that may improve the treatment of CVDs in a precision-medicine approach. Overall, as shown for COVID-19 vaccines, there is great hope that RNA constitutes the next family of RNA biomarkers for clinical practice.

## Authors’ contributions

Significant contribution to the manuscript content: M.V., R.A., M.B., S.B.-A., T.B., D.d.G.-C., C.E., R.F., J.G., M.H., B.K., F.M., M.S., S.B.W., Y.D. Manuscript drafting: M.V., Y.D. Manuscript review and revision: M.V., R.A., M.B., S.B.-A., T.B., D.d.G.-C., C.E., R.F., J.G., M.H., B.K., F.M., M.S., S.B.W., Y.D.

## References

[1] Bayes-Genis A , Voors AA, Zannad F, Januzzi JL, Mark Richards A, Diez J. Transitioning from usual care to biomarker-based personalized and precision medicine in heart failure: call for action. Eur Heart J 2018;39:2793–2799. 28204449 10.1093/eurheartj/ehx027

[2] Perrino C , Barabasi AL, Condorelli G, Davidson SM, De Windt L, Dimmeler S, Engel FB, Hausenloy DJ, Hill JA, Van Laake LW, Lecour S, Leor J, Madonna R, Mayr M, Prunier F, Sluijter JPG, Schulz R, Thum T, Ytrehus K, Ferdinandy P. Epigenomic and transcriptomic approaches in the post-genomic era: path to novel targets for diagnosis and therapy of the ischaemic heart? Position Paper of the European Society of Cardiology Working Group on Cellular Biology of the Heart. Cardiovasc Res 2017;113:725–736. 28460026 10.1093/cvr/cvx070 PMC5437366

[3] Kumarswamy R , Thum T. Non-coding RNAs in cardiac remodeling and heart failure. Circ Res 2013;113:676–689. 23989712 10.1161/CIRCRESAHA.113.300226

[4] Batkai S , Genschel C, Viereck J, Rump S, Bar C, Borchert T, Traxler D, Riesenhuber M, Spannbauer A, Lukovic D, Zlabinger K, Hasimbegovic E, Winkler J, Garamvolgyi R, Neitzel S, Gyongyosi M, Thum T. CDR132L improves systolic and diastolic function in a large animal model of chronic heart failure. Eur Heart J 2021;42:192–201. 33089304 10.1093/eurheartj/ehaa791 PMC7813625

[5] Täubel J , Hauke W, Rump S, Viereck J, Batkai S, Poetzsch J, Rode L, Weigt H, Genschel C, Lorch U, Theek C, Levin AA, Bauersachs J, Solomon SD, Thum T. Novel antisense therapy targeting microRNA-132 in patients with heart failure: results of a first-in-human Phase 1b randomized, double-blind, placebo-controlled study. Eur Heart J 2021;42:178–188. 33245749 10.1093/eurheartj/ehaa898 PMC7954267

[6] Navickas R , Gal D, Laucevičius A, Taparauskaitė A, Zdanytė M, Holvoet P. Identifying circulating microRNAs as biomarkers of cardiovascular disease: a systematic review. Cardiovasc Res 2016;111:322–337. 27357636 10.1093/cvr/cvw174 PMC4996262

[7] Schulte C , Barwari T, Joshi A, Theofilatos K, Zampetaki A, Barallobre-Barreiro J, Singh B, Sorensen NA, Neumann JT, Zeller T, Westermann D, Blankenberg S, Marber M, Liebetrau C, Mayr M. Comparative analysis of circulating noncoding RNAs versus protein biomarkers in the detection of myocardial injury. Circ Res 2019;125:328–340. 31159652 10.1161/CIRCRESAHA.119.314937 PMC6641471

[8] Biener M , Giannitsis E, Thum T, Bär C, Costa A, Andrzejewski T, Stoyanov KM, Vafaie M, Meder B, Katus HA, de Gonzalo-Calvo D, Mueller-Hennessen M. Diagnostic value of circulating microRNAs compared to high-sensitivity troponin T for the detection of non-ST-segment elevation myocardial infarction. Eur Heart J Acute Cardiovasc Care 2021;10:653–660. 33580779 10.1093/ehjacc/zuaa034

[9] Jakob P , Kacprowski T, Briand-Schumacher S, Heg D, Klingenberg R, Stähli BE, Jaguszewski M, Rodondi N, Nanchen D, Räber L, Vogt P, Mach F, Windecker S, Völker U, Matter CM, Lüscher TF, Landmesser U. Profiling and validation of circulating microRNAs for cardiovascular events in patients presenting with ST-segment elevation myocardial infarction. Eur Heart J 2017;38:511–515. 28011706 10.1093/eurheartj/ehw563

[10] Kaudewitz D , Skroblin P, Bender LH, Barwari T, Willeit P, Pechlaner R, Sunderland NP, Willeit K, Morton AC, Armstrong PC, Chan MV, Lu R, Yin X, Gracio F, Dudek K, Langley SR, Zampetaki A, de Rinaldis E, Ye S, Warner TD, Saxena A, Kiechl S, Storey RF, Mayr M. Association of MicroRNAs and YRNAs with platelet function. Circ Res 2016;118:420–432. 26646931 10.1161/CIRCRESAHA.114.305663 PMC5065093

[11] Cayla G , Cuisset T, Silvain J, Leclercq F, Manzo-Silberman S, Saint-Etienne C, Delarche N, Bellemain-Appaix A, Range G, El Mahmoud R, Carrié D, Belle L, Souteyrand G, Aubry P, Sabouret P, Du Fretay XH, Beygui F, Bonnet J-L, Lattuca B, Pouillot C, Varenne O, Boueri Z, Van Belle E, Henry P, Motreff P, Elhadad S, Salem J-E, Abtan J, Rousseau H, Collet J-P, Vicaut E, Montalescot G. Platelet function monitoring to adjust antiplatelet therapy in elderly patients stented for an acute coronary syndrome (ANTARCTIC): an open-label, blinded-endpoint, randomised controlled superiority trial. Lancet 2016;388:2015–2022. 27581531 10.1016/S0140-6736(16)31323-X

[12] Sunderland N , Skroblin P, Barwari T, Huntley RP, Lu R, Joshi A, Lovering RC, Mayr M. MicroRNA biomarkers and platelet reactivity. Circ Res 2017;120:418–435. 28104774 10.1161/CIRCRESAHA.116.309303

[13] Barrett TJ , Lee AH, Smilowitz NR, Hausvater A, Fishman GI, Hochman JS, Reynolds HR, Berger JS. Whole-blood transcriptome profiling identifies women with myocardial infarction with nonobstructive coronary artery disease. Circ Genom Precis Med 2018;11:e002387. 30562118 10.1161/CIRCGEN.118.002387 PMC6455939

[14] Elashoff MR , Wingrove JA, Beineke P, Daniels SE, Tingley WG, Rosenberg S, Voros S, Kraus WE, Ginsburg GS, Schwartz RS, Ellis SG, Tahirkheli N, Waksman R, McPherson J, Lansky AJ, Topol EJ. Development of a blood-based gene expression algorithm for assessment of obstructive coronary artery disease in non-diabetic patients. BMC Med Genomics 2011;4:26. 21443790 10.1186/1755-8794-4-26 PMC3072303

[15] Vanhaverbeke M , Vausort M, Veltman D, Zhang L, Wu M, Laenen G, Gillijns H, Moreau Y, Bartunek J, Van De Werf F, Devaux Y, Janssens S, Sinnaeve PR, Eu-Cca CA; EU-CardioRNA COST Action CA17129. Peripheral blood RNA levels of QSOX1 and PLBD1 are new independent predictors of left ventricular dysfunction after acute myocardial infarction. Circ Genom Precis Med 2019;12:e002656. 31756302 10.1161/CIRCGEN.119.002656 PMC6922070

[16] Kumarswamy R , Bauters C, Volkmann I, Maury F, Fetisch J, Holzmann A, Lemesle G, de Groote P, Pinet F, Thum T. Circulating long noncoding RNA, LIPCAR, predicts survival in patients with heart failure. Circ Res 2014;114:1569–1575. 24663402 10.1161/CIRCRESAHA.114.303915

[17] Vausort M , Salgado-Somoza A, Zhang L, Leszek P, Scholz M, Teren A, Burkhardt R, Thiery J, Wagner DR, Devaux Y. Myocardial infarction-associated circular RNA predicting left ventricular dysfunction. J Am Coll Cardiol 2016;68:1247–1248. 27609688 10.1016/j.jacc.2016.06.040

[18] Pritchard CC , Kroh E, Wood B, Arroyo JD, Dougherty KJ, Miyaji MM, Tait JF, Tewari M. Blood cell origin of circulating microRNAs: a cautionary note for cancer biomarker studies. Cancer Prev Res (Phila) 2012;5:492–497. 22158052 10.1158/1940-6207.CAPR-11-0370 PMC4186243

[19] Yu C , Li L, Xie F, Guo S, Liu F, Dong N, Wang Y. LncRNA TUG1 sponges miR-204-5p to promote osteoblast differentiation through upregulating Runx2 in aortic valve calcification. Cardiovasc Res 2018;114:168–179. 29016735 10.1093/cvr/cvx180

[20] Coffey S , Williams MJ, Phillips LV, Jones GT. Circulating microRNA profiling needs further refinement before clinical use in patients with aortic stenosis. J Am Heart Assoc 2015;4:e002150. 26304936 10.1161/JAHA.115.002150 PMC4599470

[21] McManus DD , Tanriverdi K, Lin H, Esa N, Kinno M, Mandapati D, Tam S, Okike ON, Ellinor PT, Keaney JF Jr, Donahue JK, Benjamin EJ, Freedman JE. Plasma microRNAs are associated with atrial fibrillation and change after catheter ablation (the miRhythm study). Heart Rhythm 2015;12:3–10. 25257092 10.1016/j.hrthm.2014.09.050 PMC4277933

[22] Devaux Y , Dankiewicz J, Salgado-Somoza A, Stammet P, Collignon O, Gilje P, Gidlof O, Zhang L, Vausort M, Hassager C, Wise MP, Kuiper M, Friberg H, Cronberg T, Erlinge D, Nielsen N; for Target Temperature Management After Cardiac Arrest Trial Investigators. Association of circulating microRNA-124-3p levels with outcomes after out-of-hospital cardiac arrest: a substudy of a randomized clinical trial. JAMA Cardiol 2016;1:305–313. 27438111 10.1001/jamacardio.2016.0480

[23] Blanco-Domínguez R , Sánchez-Díaz R, de la Fuente H, Jiménez-Borreguero LJ, Matesanz-Marín A, Relaño M, Jiménez-Alejandre R, Linillos-Pradillo B, Tsilingiri K, Martín-Mariscal ML, Alonso-Herranz L, Moreno G, Martín-Asenjo R, García-Guimaraes MM, Bruno KA, Dauden E, González-Álvaro I, Villar-Guimerans LM, Martínez-León A, Salvador-Garicano AM, Michelhaugh SA, Ibrahim NE, Januzzi JL, Kottwitz J, Iliceto S, Plebani M, Basso C, Baritussio A, Seguso M, Marcolongo R, Ricote M, Fairweather D, Bueno H, Fernández-Friera L, Alfonso F, Caforio ALP, Pascual-Figal DA, Heidecker B, Lüscher TF, Das S, Fuster V, Ibáñez B, Sánchez-Madrid F, Martín P. A novel circulating microRNA for the detection of acute myocarditis. N Engl J Med 2021;384:2014–2027. 34042389 10.1056/NEJMoa2003608 PMC8258773

[24] Wang K , Yuan Y, Cho JH, McClarty S, Baxter D, Galas DJ. Comparing the microRNA spectrum between serum and plasma. PLoS One 2012;7:e41561. 22859996 10.1371/journal.pone.0041561 PMC3409228

[25] Mussbacher M , Krammer TL, Heber S, Schrottmaier WC, Zeibig S, Holthoff HP, Pereyra D, Starlinger P, Hackl M, Assinger A. Impact of anticoagulation and sample processing on the quantification of human blood-derived microRNA signatures. Cells 2020;9:1915. 32824700 10.3390/cells9081915 PMC7464075

[26] Mayr M , Lee R, Kaudewitz D, Zampetaki A, Channon KM. Effects of heparin on temporal microRNA profiles. J Am Coll Cardiol 2014;63:940–941. 24315915 10.1016/j.jacc.2013.07.118 PMC5357045

[27] Kaudewitz D , Lee R, Willeit P, McGregor R, Markus HS, Kiechl S, Zampetaki A, Storey RF, Channon KM, Mayr M. Impact of intravenous heparin on quantification of circulating microRNAs in patients with coronary artery disease. Thromb Haemost 2013;110:609–615. 23803974 10.1160/TH13-05-0368

[28] Khan J , Lieberman JA, Lockwood CM. Variability in, variability out: best practice recommendations to standardize pre-analytical variables in the detection of circulating and tissue microRNAs. Clin Chem Lab Med 2017;55:608–621. 28306519 10.1515/cclm-2016-0471

[29] Mitchell PS , Parkin RK, Kroh EM, Fritz BR, Wyman SK, Pogosova-Agadjanyan EL, Peterson A, Noteboom J, O'Briant KC, Allen A, Lin DW, Urban N, Drescher CW, Knudsen BS, Stirewalt DL, Gentleman R, Vessella RL, Nelson PS, Martin DB, Tewari M. Circulating microRNAs as stable blood-based markers for cancer detection. Proc Natl Acad Sci U S A 2008;105:10513–10518. 18663219 10.1073/pnas.0804549105 PMC2492472

[30] Sourvinou IS , Markou A, Lianidou ES. Quantification of circulating miRNAs in plasma: effect of preanalytical and analytical parameters on their isolation and stability. J Mol Diagn 2013;15:827–834. 23988620 10.1016/j.jmoldx.2013.07.005

[31] Köberle V , Kakoschky B, Ibrahim AA, Schmithals C, Peveling-Oberhag J, Zeuzem S, Kronenberger B, Waidmann O, Pleli T, Piiper A. Vesicle-associated microRNAs are released from blood cells on incubation of blood samples. Transl Res 2016;169:40–46. 26608461 10.1016/j.trsl.2015.10.010

[32] Tuck MK , Chan DW, Chia D, Godwin AK, Grizzle WE, Krueger KE, Rom W, Sanda M, Sorbara L, Stass S, Wang W, Brenner DE. Standard operating procedures for serum and plasma collection: early detection research network consensus statement standard operating procedure integration working group. J Proteome Res 2009;8:113–117. 19072545 10.1021/pr800545q PMC2655764

[33] Weber DG , Casjens S, Rozynek P, Lehnert M, Zilch-Schöneweis S, Bryk O, Taeger D, Gomolka M, Kreuzer M, Otten H, Pesch B, Johnen G, Brüning T. Assessment of mRNA and microRNA stabilization in peripheral human blood for multicenter studies and biobanks. Biomark Insights 2010;5:95–102. 20981139 10.4137/bmi.s5522 PMC2956623

[34] Nair VS , Pritchard CC, Tewari M, Ioannidis JP. Design and analysis for studying microRNAs in human disease: a primer on -Omic Technologies. Am J Epidemiol 2014;180:140–152. 24966218 10.1093/aje/kwu135 PMC4082346

[35] Liu J , Walter E, Stenger D, Thach D. Effects of globin mRNA reduction methods on gene expression profiles from whole blood. J Mol Diagn 2006;8:551–558. 17065423 10.2353/jmoldx.2006.060021 PMC1876175

[36] Holvoet P , Vanhaverbeke M, Bloch K, Baatsen P, Sinnaeve P, Janssens S. Low MT-CO1 in monocytes and microvesicles is associated with outcome in patients with coronary artery disease. J Am Heart Assoc 2016;5:e004207. 27919931 10.1161/JAHA.116.004207 PMC5210432

[37] Coumans FAW , Brisson AR, Buzas EI, Dignat-George F, Drees EEE, El-Andaloussi S, Emanueli C, Gasecka A, Hendrix A, Hill AF, Lacroix R, Lee Y, van Leeuwen TG, Mackman N, Mäger I, Nolan JP, van der Pol E, Pegtel DM, Sahoo S, Siljander PRM, Sturk G, de Wever O, Nieuwland R. Methodological guidelines to study extracellular vesicles. Circ Res 2017;120:1632–1648. 28495994 10.1161/CIRCRESAHA.117.309417

[38] Chevillet JR , Kang Q, Ruf IK, Briggs HA, Vojtech LN, Hughes SM, Cheng HH, Arroyo JD, Meredith EK, Gallichotte EN, Pogosova-Agadjanyan EL, Morrissey C, Stirewalt DL, Hladik F, Yu EY, Higano CS, Tewari M. Quantitative and stoichiometric analysis of the microRNA content of exosomes. Proc Natl Acad Sci U S A 2014;111:14888–14893. 25267620 10.1073/pnas.1408301111 PMC4205618

[39] Jeppesen DK , Fenix AM, Franklin JL, Higginbotham JN, Zhang Q, Zimmerman LJ, Liebler DC, Ping J, Liu Q, Evans R, Fissell WH, Patton JG, Rome LH, Burnette DT, Coffey RJ. Reassessment of exosome composition. Cell 2019;177:428–445.e418. 30951670 10.1016/j.cell.2019.02.029 PMC6664447

[40] O’Brien K , Breyne K, Ughetto S, Laurent LC, Breakefield XO. RNA delivery by extracellular vesicles in mammalian cells and its applications. Nat Rev Mol Cell Biol 2020;21:585–606. 32457507 10.1038/s41580-020-0251-y PMC7249041

[41] Théry C , Witwer KW, Aikawa E, Alcaraz MJ, Anderson JD, Andriantsitohaina R, Antoniou A, Arab T, Archer F, Atkin-Smith GK, Ayre DC, Bach JM, Bachurski D, Baharvand H, Balaj L, Baldacchino S, Bauer NN, Baxter AA, Bebawy M, Beckham C, Bedina Zavec A, Benmoussa A, Berardi AC, Bergese P, Bielska E, Blenkiron C, Bobis-Wozowicz S, Boilard E, Boireau W, Bongiovanni A, Borràs FE, Bosch S, Boulanger CM, Breakefield X, Breglio AM, Brennan MÁ, Brigstock DR, Brisson A, Broekman ML, Bromberg JF, Bryl-Górecka P, Buch S, Buck AH, Burger D, Busatto S, Buschmann D, Bussolati B, Buzás EI, Byrd JB, Camussi G, Carter DR, Caruso S, Chamley LW, Chang YT, Chen C, Chen S, Cheng L, Chin AR, Clayton A, Clerici SP, Cocks A, Cocucci E, Coffey RJ, Cordeiro-da-Silva A, Couch Y, Coumans FA, Coyle B, Crescitelli R, Criado MF, D'Souza-Schorey C, Das S, Datta Chaudhuri A, de Candia P, De Santana EF, De Wever O, Del Portillo HA, Demaret T, Deville S, Devitt A, Dhondt B, Di Vizio D, Dieterich LC, Dolo V, Dominguez Rubio AP, Dominici M, Dourado MR, Driedonks TA, Duarte FV, Duncan HM, Eichenberger RM, Ekström K, El Andaloussi S, Elie-Caille C, Erdbrügger U, Falcón-Pérez JM, Fatima F, Fish JE, Flores-Bellver M, Försönits A, Frelet-Barrand A, Fricke F, Fuhrmann G, Gabrielsson S, Gámez-Valero A, Gardiner C, Gärtner K, Gaudin R, Gho YS, Giebel B, Gilbert C, Gimona M, Giusti I, Goberdhan DC, Görgens A, Gorski SM, Greening DW, Gross JC, Gualerzi A, Gupta GN, Gustafson D, Handberg A, Haraszti RA, Harrison P, Hegyesi H, Hendrix A, Hill AF, Hochberg FH, Hoffmann KF, Holder B, Holthofer H, Hosseinkhani B, Hu G, Huang Y, Huber V, Hunt S, Ibrahim AG, Ikezu T, Inal JM, Isin M, Ivanova A, Jackson HK, Jacobsen S, Jay SM, Jayachandran M, Jenster G, Jiang L, Johnson SM, Jones JC, Jong A, Jovanovic-Talisman T, Jung S, Kalluri R, Kano SI, Kaur S, Kawamura Y, Keller ET, Khamari D, Khomyakova E, Khvorova A, Kierulf P, Kim KP, Kislinger T, Klingeborn M, Klinke DJ 2nd, Kornek M, Kosanović MM, Kovács ÁF, Krämer-Albers EM, Krasemann S, Krause M, Kurochkin IV, Kusuma GD, Kuypers S, Laitinen S, Langevin SM, Languino LR, Lannigan J, Lässer C, Laurent LC, Lavieu G, Lázaro-Ibáñez E, Le Lay S, Lee MS, Lee YXF, Lemos DS, Lenassi M, Leszczynska A, Li IT, Liao K, Libregts SF, Ligeti E, Lim R, Lim SK, Linē A, Linnemannstöns K, Llorente A, Lombard CA, Lorenowicz MJ, Lörincz ÁM, Lötvall J, Lovett J, Lowry MC, Loyer X, Lu Q, Lukomska B, Lunavat TR, Maas SL, Malhi H, Marcilla A, Mariani J, Mariscal J, Martens-Uzunova ES, Martin-Jaular L, Martinez MC, Martins VR, Mathieu M, Mathivanan S, Maugeri M, McGinnis LK, McVey MJ, Meckes DG Jr, Meehan KL, Mertens I, Minciacchi VR, Möller A, Møller Jørgensen M, Morales-Kastresana A, Morhayim J, Mullier F, Muraca M, Musante L, Mussack V, Muth DC, Myburgh KH, Najrana T, Nawaz M, Nazarenko I, Nejsum P, Neri C, Neri T, Nieuwland R, Nimrichter L, Nolan JP, Nolte-'t Hoen EN, Noren Hooten N, O'Driscoll L, O'Grady T, O'Loghlen A, Ochiya T, Olivier M, Ortiz A, Ortiz LA, Osteikoetxea X, Østergaard O, Ostrowski M, Park J, Pegtel DM, Peinado H, Perut F, Pfaffl MW, Phinney DG, Pieters BC, Pink RC, Pisetsky DS, Pogge von Strandmann E, Polakovicova I, Poon IK, Powell BH, Prada I, Pulliam L, Quesenberry P, Radeghieri A, Raffai RL, Raimondo S, Rak J, Ramirez MI, Raposo G, Rayyan MS, Regev-Rudzki N, Ricklefs FL, Robbins PD, Roberts DD, Rodrigues SC, Rohde E, Rome S, Rouschop KM, Rughetti A, Russell AE, Saá P, Sahoo S, Salas-Huenuleo E, Sánchez C, Saugstad JA, Saul MJ, Schiffelers RM, Schneider R, Schøyen TH, Scott A, Shahaj E, Sharma S, Shatnyeva O, Shekari F, Shelke GV, Shetty AK, Shiba K, Siljander PR, Silva AM, Skowronek A, Snyder OL 2nd, Soares RP, Sódar BW, Soekmadji C, Sotillo J, Stahl PD, Stoorvogel W, Stott SL, Strasser EF, Swift S, Tahara H, Tewari M, Timms K, Tiwari S, Tixeira R, Tkach M, Toh WS, Tomasini R, Torrecilhas AC, Tosar JP, Toxavidis V, Urbanelli L, Vader P, van Balkom BW, van der Grein SG, Van Deun J, van Herwijnen MJ, Van Keuren-Jensen K, van Niel G, van Royen ME, van Wijnen AJ, Vasconcelos MH, Vechetti IJ Jr, Veit TD, Vella LJ, Velot É, Verweij FJ, Vestad B, Viñas JL, Visnovitz T, Vukman KV, Wahlgren J, Watson DC, Wauben MH, Weaver A, Webber JP, Weber V, Wehman AM, Weiss DJ, Welsh JA, Wendt S, Wheelock AM, Wiener Z, Witte L, Wolfram J, Xagorari A, Xander P, Xu J, Yan X, Yáñez-Mó M, Yin H, Yuana Y, Zappulli V, Zarubova J, Žėkas V, Zhang JY, Zhao Z, Zheng L, Zheutlin AR, Zickler AM, Zimmermann P, Zivkovic AM, Zocco D, Zuba-Surma EK. Minimal information for studies of extracellular vesicles 2018 (MISEV2018): a position statement of the International Society for Extracellular Vesicles and update of the MISEV2014 guidelines. J Extracell Vesicles 2018;7:1535750. 30637094 10.1080/20013078.2018.1535750 PMC6322352

[42] Chiva-Blanch G , Bratseth V, Ritschel V, Andersen GO, Halvorsen S, Eritsland J, Arnesen H, Badimon L, Seljeflot I. Monocyte-derived circulating microparticles (CD14(+), CD14(+)/CD11b(+) and CD14(+)/CD142(+)) are related to long-term prognosis for cardiovascular mortality in STEMI patients. Int J Cardiol 2017;227:876–881. 27915085 10.1016/j.ijcard.2016.11.302

[43] Li X , Mauro M, Williams Z. Comparison of plasma extracellular RNA isolation kits reveals kit-dependent biases. Biotechniques 2015;59:13–17. 26156779 10.2144/000114306

[44] Hackl M , Semmelrock E, Grillari J. Analytical challenges in microRNA biomarker development: best practices for analyzing microRNAs in cell-free biofluids. In: Epigenetics in Cardiovascular Disease, Elsevier, 2021. p415–430.

[45] Schwarzenbach H , da Silva AM, Calin G, Pantel K. Data normalization strategies for microRNA quantification. Clin Chem 2015;61:1333–1342. 26408530 10.1373/clinchem.2015.239459 PMC4890630

[46] Robinson EL , Baker AH, Brittan M, McCracken I, Condorelli G, Emanueli C, Srivastava KP, Gaetano C, Thum T, Vanhaverbeke M, Angione C, Heymans S, Devaux Y, Pedrazzini T, Martelli F; EU-CardioRNA COST Action CA17129. Dissecting the transcriptome in cardiovascular disease. Cardiovasc Res 2022;118:1004–1019. 33757121 10.1093/cvr/cvab117 PMC8930073

[47] Stark R , Grzelak M, Hadfield J. RNA sequencing: the teenage years. Nat Rev Genet 2019;20:631–656. 31341269 10.1038/s41576-019-0150-2

[48] Shore S , Henderson JM, Lebedev A, Salcedo MP, Zon G, McCaffrey AP, Paul N, Hogrefe RI. Small RNA library preparation method for next-generation sequencing using chemical modifications to prevent adapter dimer formation. PLoS One 2016;11:e0167009. 27875576 10.1371/journal.pone.0167009 PMC5119831

[49] Barberán-Soler S , Vo JM, Hogans RE, Dallas A, Johnston BH, Kazakov SA. Decreasing miRNA sequencing bias using a single adapter and circularization approach. Genome Biol 2018;19:105. 30173660 10.1186/s13059-018-1488-z PMC6120088

[50] Fu Y , Wu P-H, Beane T, Zamore PD, Weng Z. Elimination of PCR duplicates in RNA-seq and small RNA-seq using unique molecular identifiers. BMC Genomics 2018;19:531. 30001700 10.1186/s12864-018-4933-1 PMC6044086

[51] Donati S , Ciuffi S, Brandi ML. Human circulating miRNAs real-time qRT-PCR-based analysis: an overview of endogenous reference genes used for data normalization. Int J Mol Sci 2019;20:4353. 31491899 10.3390/ijms20184353 PMC6769746

[52] Heegaard NHH , Carlsen AL, Lilje B, Ng KL, Rønne ME, Jørgensen HL, Sennels H, Fahrenkrug J. Diurnal variations of human circulating cell-free micro-RNA. PLoS One 2016;11:e0160577. 27494182 10.1371/journal.pone.0160577 PMC4975411

[53] De Boever P , Wens B, Forcheh AC, Reynders H, Nelen V, Kleinjans J, Van Larebeke N, Verbeke G, Valkenborg D, Schoeters G. Characterization of the peripheral blood transcriptome in a repeated measures design using a panel of healthy individuals. Genomics 2014;103:31–39. 24321174 10.1016/j.ygeno.2013.11.006

[54] Chilton WL , Marques FZ, West J, Kannourakis G, Berzins SP, O’Brien BJ, Charchar FJ. Acute exercise leads to regulation of telomere-associated genes and microRNA expression in immune cells. PLoS One 2014;9:e92088. 24752326 10.1371/journal.pone.0092088 PMC3994003

[55] Barber JL , Zellars KN, Barringhaus KG, Bouchard C, Spinale FG, Sarzynski MA. The effects of regular exercise on circulating cardiovascular-related microRNAs. Sci Rep 2019;9:7527. 31101833 10.1038/s41598-019-43978-x PMC6525243

[56] de Gonzalo-Calvo D , Dávalos A, Fernández-Sanjurjo M, Amado-Rodríguez L, Díaz-Coto S, Tomás-Zapico C, Montero A, García-González Á, Llorente-Cortés V, Heras ME, Boraita Pérez A, Díaz-Martínez ÁE, Úbeda N, Iglesias-Gutiérrez E. Circulating microRNAs as emerging cardiac biomarkers responsive to acute exercise. Int J Cardiol 2018;264:130–136. 29776561 10.1016/j.ijcard.2018.02.092

[57] Keller A , Rounge T, Backes C, Ludwig N, Gislefoss R, Leidinger P, Langseth H, Meese E. Sources to variability in circulating human miRNA signatures. RNA Biol 2017;14:1791–1798. 28820329 10.1080/15476286.2017.1367888 PMC5731815

[58] Rounge TB , Umu SU, Keller A, Meese E, Ursin G, Tretli S, Lyle R, Langseth H. Circulating small non-coding RNAs associated with age, sex, smoking, body mass and physical activity. Sci Rep 2018;8:17650. 30518766 10.1038/s41598-018-35974-4 PMC6281647

[59] Peters MJ , Joehanes R, Pilling LC, Schurmann C, Conneely KN, Powell J, Reinmaa E, Sutphin GL, Zhernakova A, Schramm K, Wilson YA, Kobes S, Tukiainen T, Ramos YF, Göring HHH, Fornage M, Liu Y, Gharib SA, Stranger BE, De Jager PL, Aviv A, Levy D, Murabito JM, Munson PJ, Huan T, Hofman A, Uitterlinden AG, Rivadeneira F, van Rooij J, Stolk L, Broer L, Verbiest MMPJ, Jhamai M, Arp P, Metspalu A, Tserel L, Milani L, Samani NJ, Peterson P, Kasela S, Codd V, Peters A, Ward-Caviness CK, Herder C, Waldenberger M, Roden M, Singmann P, Zeilinger S, Illig T, Homuth G, Grabe H-J, Völzke H, Steil L, Kocher T, Murray A, Melzer D, Yaghootkar H, Bandinelli S, Moses EK, Kent JW, Curran JE, Johnson MP, Williams-Blangero S, Westra H-J, McRae AF, Smith JA, Kardia SLR, Hovatta I, Perola M, Ripatti S, Salomaa V, Henders AK, Martin NG, Smith AK, Mehta D, Binder EB, Nylocks KM, Kennedy EM, Klengel T, Ding J, Suchy-Dicey AM, Enquobahrie DA, Brody J, Rotter JI, Chen Y-DI, Houwing-Duistermaat J, Kloppenburg M, Slagboom PE, Helmer Q, den Hollander W, Bean S, Raj T, Bakhshi N, Wang QP, Oyston LJ, Psaty BM, Tracy RP, Montgomery GW, Turner ST, Blangero J, Meulenbelt I, Ressler KJ, Yang J, Franke L, Kettunen J, Visscher PM, Neely GG, Korstanje R, Hanson RL, Prokisch H, Ferrucci L, Esko T, Teumer A, van Meurs JBJ, Johnson AD; NABEC/UKBEC Consortium. The transcriptional landscape of age in human peripheral blood. Nat Commun 2015;6:8570. 26490707 10.1038/ncomms9570 PMC4639797

[60] Huan T , Chen G, Liu C, Bhattacharya A, Rong J, Chen BH, Seshadri S, Tanriverdi K, Freedman JE, Larson MG, Murabito JM, Levy D. Age-associated microRNA expression in human peripheral blood is associated with all-cause mortality and age-related traits. Aging Cell 2018;17:e12687. 29044988 10.1111/acel.12687 PMC5770777

[61] Lopes-Ramos CM , Chen C-Y, Kuijjer ML, Paulson JN, Sonawane AR, Fagny M, Platig J, Glass K, Quackenbush J, DeMeo DL. Sex differences in gene expression and regulatory networks across 29 human tissues. Cell Reports 2020;31:107795. 32579922 10.1016/j.celrep.2020.107795 PMC7898458

[62] Thomas GS , Voros S, McPherson JA, Lansky AJ, Winn ME, Bateman TM, Elashoff MR, Lieu HD, Johnson AM, Daniels SE, Ladapo JA, Phelps CE, Douglas PS, Rosenberg S. A blood-based gene expression test for obstructive coronary artery disease tested in symptomatic nondiabetic patients referred for myocardial perfusion imaging the COMPASS study. Circ Cardiovasc Genet 2013;6:154–162. 23418288 10.1161/CIRCGENETICS.112.964015

[63] Eisenberg I , Nahmias N, Novoselsky Persky M, Greenfield C, Goldman-Wohl D, Hurwitz A, Haimov-Kochman R, Yagel S, Imbar T. Elevated circulating micro-ribonucleic acid (miRNA)-200b and miRNA-429 levels in anovulatory women. Fertil Steril 2017;107:269–275. 27816236 10.1016/j.fertnstert.2016.10.003

[64] Charlesworth JC , Curran JE, Johnson MP, Göring HH, Dyer TD, Diego VP, Kent JW Jr, Mahaney MC, Almasy L, MacCluer JW, Moses EK, Blangero J. Transcriptomic epidemiology of smoking: the effect of smoking on gene expression in lymphocytes. BMC Med Genomics 2010;3:29. 20633249 10.1186/1755-8794-3-29 PMC2911391

[65] Vink JM , Jansen R, Brooks A, Willemsen G, van Grootheest G, de Geus E, Smit JH, Penninx BW, Boomsma DI. Differential gene expression patterns between smokers and non-smokers: cause or consequence? Addict Biol 2017;22:550–560. 26594007 10.1111/adb.12322 PMC5347870

[66] Grayson BL , Wang L, Aune TM. Peripheral blood gene expression profiles in metabolic syndrome, coronary artery disease and type 2 diabetes. Genes Immun 2011;12:341–351. 21368773 10.1038/gene.2011.13 PMC3137736

[67] Hulsmans M , Sinnaeve P, Van der Schueren B, Mathieu C, Janssens S, Holvoet P. Decreased miR-181a expression in monocytes of obese patients is associated with the occurrence of metabolic syndrome and coronary artery disease. J Clin Endocrinol Metab 2012;97:E1213–E1218. 22535975 10.1210/jc.2012-1008

[68] Huan T , Esko T, Peters MJ, Pilling LC, Schramm K, Schurmann C, Chen BH, Liu C, Joehanes R, Johnson AD, Yao C, Ying SX, Courchesne P, Milani L, Raghavachari N, Wang R, Liu P, Reinmaa E, Dehghan A, Hofman A, Uitterlinden AG, Hernandez DG, Bandinelli S, Singleton A, Melzer D, Metspalu A, Carstensen M, Grallert H, Herder C, Meitinger T, Peters A, Roden M, Waldenberger M, Dorr M, Felix SB, Zeller T, International Consortium For Blood Pressure G, Vasan R, O’Donnell CJ, Munson PJ, Yang X, Prokisch H, Volker U, van Meurs JB, Ferrucci L, Levy D; International Consortium for Blood Pressure GWAS (ICBP). A meta-analysis of gene expression signatures of blood pressure and hypertension. PLoS Genet 2015;11:e1005035. 25785607 10.1371/journal.pgen.1005035 PMC4365001

[69] Bigler J , Boedigheimer M, Schofield JPR, Skipp PJ, Corfield J, Rowe A, Sousa AR, Timour M, Twehues L, Hu X, Roberts G, Welcher AA, Yu W, Lefaudeux D, Meulder B, Auffray C, Chung KF, Adcock IM, Sterk PJ, Djukanovic R, Group UBS; U-BIOPRED Study Group. A severe asthma disease signature from gene expression profiling of peripheral blood from U-BIOPRED cohorts. Am J Respir Crit Care Med 2017;195:1311–1320. 27925796 10.1164/rccm.201604-0866OC

[70] Leidinger P , Keller A, Borries A, Huwer H, Rohling M, Huebers J, Lenhof HP, Meese E. Specific peripheral miRNA profiles for distinguishing lung cancer from COPD. Lung Cancer 2011;74:41–47. 21388703 10.1016/j.lungcan.2011.02.003

[71] Abbas AR , Wolslegel K, Seshasayee D, Modrusan Z, Clark HF. Deconvolution of blood microarray data identifies cellular activation patterns in systemic lupus erythematosus. PLoS One 2009;4:e6098. 19568420 10.1371/journal.pone.0006098 PMC2699551

[72] Pimentel-Santos FM , Ligeiro D, Matos M, Mourão AF, Costa J, Santos H, Barcelos A, Godinho F, Pinto P, Cruz M, Fonseca JE, Guedes-Pinto H, Branco JC, Brown MA, Thomas GP. Whole blood transcriptional profiling in ankylosing spondylitis identifies novel candidate genes that might contribute to the inflammatory and tissue-destructive disease aspects. Arthritis Res Ther 2011;13:R57. 21470430 10.1186/ar3309 PMC3132052

[73] Anfossi S , Babayan A, Pantel K, Calin GA. Clinical utility of circulating non-coding RNAs — an update. Nat Rev Clin Oncol 2018;15:541–563. 29784926 10.1038/s41571-018-0035-x

[74] Kurian SM , Williams AN, Gelbart T, Campbell D, Mondala TS, Head SR, Horvath S, Gaber L, Thompson R, Whisenant T, Lin W, Langfelder P, Robison EH, Schaffer RL, Fisher JS, Friedewald J, Flechner SM, Chan LK, Wiseman AC, Shidban H, Mendez R, Heilman R, Abecassis MM, Marsh CL, Salomon DR. Molecular classifiers for acute kidney transplant rejection in peripheral blood by whole genome gene expression profiling. Am J Transplant 2014;14:1164–1172. 24725967 10.1111/ajt.12671 PMC4439107

[75] Lin H , Yin X, Lunetta KL, Dupuis J, McManus DD, Lubitz SA, Magnani JW, Joehanes R, Munson PJ, Larson MG, Levy D, Ellinor PT, Benjamin EJ. Whole blood gene expression and atrial fibrillation: the Framingham Heart Study. PLoS One 2014;9:e96794. 24805109 10.1371/journal.pone.0096794 PMC4013062

[76] de Gonzalo-Calvo D , Kenneweg F, Bang C, Toro R, van der Meer RW, Rijzewijk LJ, Smit JW, Lamb HJ, Llorente-Cortes V, Thum T. Circulating long noncoding RNAs in personalized medicine: response to pioglitazone therapy in type 2 diabetes. J Am Coll Cardiol 2016;68:2914–2916. 28007154 10.1016/j.jacc.2016.10.014

[77] Tijsen AJ , Creemers EE, Moerland PD, de Windt LJ, van der Wal AC, Kok WE, Pinto YM. MiR423-5p as a circulating biomarker for heart failure. Circ Res 2010;106:1035–1039. 20185794 10.1161/CIRCRESAHA.110.218297

[78] Fichtlscherer S , De Rosa S, Fox H, Schwietz T, Fischer A, Liebetrau C, Weber M, Hamm CW, Roxe T, Muller-Ardogan M, Bonauer A, Zeiher AM, Dimmeler S. Circulating microRNAs in patients with coronary artery disease. Circ Res 2010;107:677–684. 20595655 10.1161/CIRCRESAHA.109.215566

[79] Cai Y , Yang Y, Chen X, Wu G, Zhang X, Liu Y, Yu J, Wang X, Fu J, Li C, Jose PA, Zeng C, Zhou L. Circulating ‘lncRNA OTTHUMT00000387022’ from monocytes as a novel biomarker for coronary artery disease. Cardiovasc Res 2016;112:714–724. 26857419 10.1093/cvr/cvw022

[80] Røsjø H , Dahl MB, Bye A, Andreassen J, Jørgensen M, Wisløff U, Christensen G, Edvardsen T, Omland T. Prognostic value of circulating microRNA-210 levels in patients with moderate to severe aortic stenosis. PLoS One 2014;9:e91812. 24626394 10.1371/journal.pone.0091812 PMC3953554

[81] Obeidat M , Fishbane N, Nie Y, Chen V, Hollander Z, Tebbutt SJ, Bossé Y, Ng RT, Miller BE, McManus B, Rennard S, Paré PD, Sin DD. The effect of statins on blood gene expression in COPD. PLoS One 2015;10:e0140022. 26462087 10.1371/journal.pone.0140022 PMC4604084

[82] de Gonzalo-Calvo D , Vea A, Bar C, Fiedler J, Couch LS, Brotons C, Llorente-Cortes V, Thum T. Circulating non-coding RNAs in biomarker-guided cardiovascular therapy: a novel tool for personalized medicine? Eur Heart J 2019;40:1643–1650. 29688487 10.1093/eurheartj/ehy234 PMC6528150

[83] Willeit P , Zampetaki A, Dudek K, Kaudewitz D, King A, Kirkby NS, Crosby-Nwaobi R, Prokopi M, Drozdov I, Langley SR, Sivaprasad S, Markus HS, Mitchell JA, Warner TD, Kiechl S, Mayr M. Circulating microRNAs as novel biomarkers for platelet activation. Circ Res 2013;112:595–600. 23283721 10.1161/CIRCRESAHA.111.300539

[84] Deng MC , Eisen HJ, Mehra MR, Billingham M, Marboe CC, Berry G, Kobashigawa J, Johnson FL, Starling RC, Murali S, Pauly DF, Baron H, Wohlgemuth JG, Woodward RN, Klingler TM, Walther D, Lal PG, Rosenberg S, Hunt S, Investigators C; CARGO Investigators. Noninvasive discrimination of rejection in cardiac allograft recipients using gene expression profiling. Am J Transplant 2006;6:150–160. 16433769 10.1111/j.1600-6143.2005.01175.x

[85] Barr TL , Conley Y, Ding J, Dillman A, Warach S, Singleton A, Matarin M. Genomic biomarkers and cellular pathways of ischemic stroke by RNA gene expression profiling. Neurology 2010;75:1009–1014. 20837969 10.1212/WNL.0b013e3181f2b37f PMC2942033

[86] Sweeney TE , Azad TD, Donato M, Haynes WA, Perumal TM, Henao R, Bermejo-Martin JF, Almansa R, Tamayo E, Howrylak JA, Choi A, Parnell GP, Tang B, Nichols M, Woods CW, Ginsburg GS, Kingsmore SF, Omberg L, Mangravite LM, Wong HR, Tsalik EL, Langley RJ, Khatri P. Unsupervised analysis of transcriptomics in bacterial sepsis across multiple datasets reveals three robust clusters. Crit Care Med 2018;46:915–925. 29537985 10.1097/CCM.0000000000003084 PMC5953807

[87] Khalid O , Kim JJ, Kim HS, Hoang M, Tu TG, Elie O, Lee C, Vu C, Horvath S, Spigelman I, Kim Y. Gene expression signatures affected by alcohol-induced DNA methylomic deregulation in human embryonic stem cells. Stem Cell Res 2014;12:791–806. 24751885 10.1016/j.scr.2014.03.009 PMC4041389

[88] Max KEA , Bertram K, Akat KM, Bogardus KA, Li J, Morozov P, Ben-Dov IZ, Li X, Weiss ZR, Azizian A, Sopeyin A, Diacovo TG, Adamidi C, Williams Z, Tuschl T. Human plasma and serum extracellular small RNA reference profiles and their clinical utility. Proc Natl Acad Sci U S A 2018;115:E5334–E5343. 29777089 10.1073/pnas.1714397115 PMC6003356

[89] Kogure A , Uno M, Ikeda T, Nishida E. The microRNA machinery regulates fasting-induced changes in gene expression and longevity in *Caenorhabditis elegans*. J Biol Chem 2017;292:11300–11309. 28507100 10.1074/jbc.M116.765065 PMC5500796

[90] Quintanilha BJ , Reis BZ, Duarte GBS, Cozzolino SMF, Rogero MM. Nutrimiromics: role of microRNAs and nutrition in modulating inflammation and chronic diseases. Nutrients 2017;9:1168. 29077020 10.3390/nu9111168 PMC5707640

[91] Lemay DG , Huang S, Huang L, Alkan Z, Kirschke C, Burnett DJ, Wang YE, Hwang DH. Temporal changes in postprandial blood transcriptomes reveal subject-specific pattern of expression of innate immunity genes after a high-fat meal. J Nutr Biochem 2019;72:108209. 31473510 10.1016/j.jnutbio.2019.06.007

[92] Bouchard-Mercier A , Paradis AM, Rudkowska I, Lemieux S, Couture P, Vohl MC. Associations between dietary patterns and gene expression profiles of healthy men and women: a cross-sectional study. Nutr J 2013;12:24. 23398686 10.1186/1475-2891-12-24 PMC3598224

[93] Dopico XC , Evangelou M, Ferreira RC, Guo H, Pekalski ML, Smyth DJ, Cooper N, Burren OS, Fulford AJ, Hennig BJ, Prentice AM, Ziegler AG, Bonifacio E, Wallace C, Todd JA. Widespread seasonal gene expression reveals annual differences in human immunity and physiology. Nat Commun 2015;6:7000. 25965853 10.1038/ncomms8000 PMC4432600

[94] Ludwig N , Hecksteden A, Kahraman M, Fehlmann T, Laufer T, Kern F, Meyer T, Meese E, Keller A, Backes C. Spring is in the air: seasonal profiles indicate vernal change of miRNA activity. RNA Biol 2019;16:1034–1043. 31035857 10.1080/15476286.2019.1612217 PMC6602417

[95] Kinoshita C , Aoyama K, Matsumura N, Kikuchi-Utsumi K, Watabe M, Nakaki T. Rhythmic oscillations of the microRNA miR-96-5p play a neuroprotective role by indirectly regulating glutathione levels. Nat Commun 2014;5:3823. 24804999 10.1038/ncomms4823 PMC4024755

[96] Yan Y , Salazar TE, Dominguez JM 2nd, Nguyen DV, Li Calzi S, Bhatwadekar AD, Qi X, Busik JV, Boulton ME, Grant MB. Dicer expression exhibits a tissue-specific diurnal pattern that is lost during aging and in diabetes. PLoS One 2013;8:e80029. 24244599 10.1371/journal.pone.0080029 PMC3820540

[97] Mooney C , Raoof R, El-Naggar H, Sanz-Rodriguez A, Jimenez-Mateos EM, Henshall DC. High throughput qPCR expression profiling of circulating microRNAs reveals minimal sex- and sample timing-related variation in plasma of healthy volunteers. PLoS One 2015;10:e0145316. 26699132 10.1371/journal.pone.0145316 PMC4689368

[98] Ioannidis J , Donadeu FX. Circulating microRNA profiles during the bovine oestrous cycle. PLoS One 2016;11:e0158160. 27340826 10.1371/journal.pone.0158160 PMC4920432

[99] Rekker K , Saare M, Roost AM, Salumets A, Peters M. Circulating microRNA profile throughout the menstrual cycle. PLoS One 2013;8:e81166. 24244734 10.1371/journal.pone.0081166 PMC3828277

[100] McManus DD , Rong J, Huan T, Lacey S, Tanriverdi K, Munson PJ, Larson MG, Joehanes R, Murthy V, Shah R, Freedman JE, Levy D. Messenger RNA and MicroRNA transcriptomic signatures of cardiometabolic risk factors. BMC Genomics 2017;18:139. 28178938 10.1186/s12864-017-3533-9 PMC5299677

[101] Ghosh S , Dent R, Harper ME, Gorman SA, Stuart JS, McPherson R. Gene expression profiling in whole blood identifies distinct biological pathways associated with obesity. BMC Med Genomics 2010;3:56. 21122113 10.1186/1755-8794-3-56 PMC3014865

[102] Christodoulou MI , Avgeris M, Kokkinopoulou I, Maratou E, Mitrou P, Kontos CK, Pappas E, Boutati E, Scorilas A, Fragoulis EG. Blood-based analysis of type-2 diabetes mellitus susceptibility genes identifies specific transcript variants with deregulated expression and association with disease risk. Sci Rep 2019;9:1512. 30728419 10.1038/s41598-018-37856-1 PMC6365563

[103] Jongstra-Bilen J , Zhang CX, Wisnicki T, Li MK, White-Alfred S, Ilaalagan R, Ferri DM, Deonarain A, Wan MH, Hyduk SJ, Cummins CL, Cybulsky MI. Oxidized low-density lipoprotein loading of macrophages downregulates TLR-induced proinflammatory responses in a gene-specific and temporal manner through transcriptional control. J Immunol 2017;199:2149–2157. 28784845 10.4049/jimmunol.1601363

[104] Wei P , Lu Z, Song J. Variable importance analysis: a comprehensive review. Reliab Eng Syst Saf 2015;142:399–432.

[105] Zuber V , Strimmer K. High-dimensional regression and variable selection using CAR scores. Stat Appl Genet Mol Biol 2011;10: doi:10.2202/1544-6115.1730.

[106] Völzke H , Schmidt CO, Baumeister SE, Ittermann T, Fung G, Krafczyk-Korth J, Hoffmann W, Schwab M, Meyer zu Schwabedissen HE, Dörr M, Felix SB, Lieb W, Kroemer HK. Personalized cardiovascular medicine: concepts and methodological considerations. Nat Rev Cardiol 2013;10:308–316. 23528962 10.1038/nrcardio.2013.35

[107] Hausser J , Strimmer K. Entropy inference and the James-Stein estimator, with application to nonlinear gene association networks. J Mach Learn Res 2009;10:1469–1484.

[108] Arlot S , Celisse A. A survey of cross-validation procedures for model selection. Statist Surv 2010;4:40–79.

[109] Efron B , Tibshirani R. Improvements on cross-validation: the 632+ bootstrap method. J Am Stat Assoc 1997;92:548–560.

[110] Harrell FE Jr , Lee KL, Mark DB. Multivariable prognostic models: issues in developing models, evaluating assumptions and adequacy, and measuring and reducing errors. Stat Med 1996;15:361–387. 8668867 10.1002/(SICI)1097-0258(19960229)15:4<361::AID-SIM168>3.0.CO;2-4

[111] Leening MJ , Vedder MM, Witteman JC, Pencina MJ, Steyerberg EW. Net reclassification improvement: computation, interpretation, and controversies: a literature review and clinician’s guide. Ann Intern Med 2014;160:122–131. 24592497 10.7326/M13-1522

[112] Kerr KF , Wang Z, Janes H, McClelland RL, Psaty BM, Pepe MS. Net reclassification indices for evaluating risk prediction instruments: a critical review. Epidemiology 2014;25:114–121. 24240655 10.1097/EDE.0000000000000018 PMC3918180

[113] Pencina MJ , D’Agostino RB Sr, D’Agostino RB Jr, Vasan RS. Evaluating the added predictive ability of a new marker: from area under the ROC curve to reclassification and beyond. Stat Med 2008;27:157–172; discussion 207–112. 17569110 10.1002/sim.2929

[114] Burch PM , Glaab WE, Holder DJ, Phillips JA, Sauer JM, Walker EG. Net reclassification index and integrated discrimination index are not appropriate for testing whether a biomarker improves predictive performance. Toxicol Sci 2017;156:11–13. 27815493 10.1093/toxsci/kfw225 PMC5837334

[115] Rembold CM. Number needed to screen: development of a statistic for disease screening. BMJ 1998;317:307–312. 9685274 10.1136/bmj.317.7154.307 PMC28622

[116] Dagher G , Becker KF, Bonin S, Foy C, Gelmini S, Kubista M, Kungl P, Oelmueller U, Parkes H, Pinzani P, Riegman P, Schröder U, Stumptner C, Turano P, Sjöback R, Wutte A, Zatloukal K. Pre-analytical processes in medical diagnostics: new regulatory requirements and standards. N Biotechnol 2019;52:121–125. 31102798 10.1016/j.nbt.2019.05.002

[117] Fabri-Faja N , Calvo-Lozano O, Dey P, Terborg RA, Estevez MC, Belushkin A, Yesilköy F, Duempelmann L, Altug H, Pruneri V, Lechuga LM. Early sepsis diagnosis via protein and miRNA biomarkers using a novel point-of-care photonic biosensor. Anal Chim Acta 2019;1077:232–242. 31307714 10.1016/j.aca.2019.05.038

[118] Ladang A , Beaudart C, Locquet M, Reginster JY, Bruyère O, Cavalier E. Evaluation of a panel of microRNAs that predicts fragility fracture risk: a pilot study. Calcif Tissue Int 2020;106:239–247. 31729554 10.1007/s00223-019-00628-8

[119] Walter E , Dellago H, Grillari J, Dimai HP, Hackl M. Cost-utility analysis of fracture risk assessment using microRNAs compared with standard tools and no monitoring in the Austrian female population. Bone 2018;108:44–54. 29269173 10.1016/j.bone.2017.12.017

[120] Rosenberg S , Elashoff MR, Beineke P, Daniels SE, Wingrove JA, Tingley WG, Sager PT, Sehnert AJ, Yau M, Kraus WE, Newby LK, Schwartz RS, Voros S, Ellis SG, Tahirkheli N, Waksman R, McPherson J, Lansky A, Winn ME, Schork NJ, Topol EJ. Multicenter validation of the diagnostic accuracy of a blood-based gene expression test for assessing obstructive coronary artery disease in nondiabetic patients. Ann Intern Med 2010;153:425–434. 20921541 10.7326/0003-4819-153-7-201010050-00005 PMC3786733

[121] Pham MX , Teuteberg JJ, Kfoury AG, Starling RC, Deng MC, Cappola TP, Kao A, Anderson AS, Cotts WG, Ewald GA, Baran DA, Bogaev RC, Elashoff B, Baron H, Yee J, Valantine HA; IMAGE Study Group. Gene-expression profiling for rejection surveillance after cardiac transplantation. N Engl J Med 2010;362:1890–1900. 20413602 10.1056/NEJMoa0912965

[122] Crespo-Leiro MG , Stypmann J, Schulz U, Zuckermann A, Mohacsi P, Bara C, Ross H, Parameshwar J, Zakliczyński M, Fiocchi R, Hoefer D, Colvin M, Deng MC, Leprince P, Elashoff B, Yee JP, Vanhaecke J. Clinical usefulness of gene-expression profile to rule out acute rejection after heart transplantation: CARGO II. Eur Heart J 2016;37:2591–2601. 26746629 10.1093/eurheartj/ehv682 PMC5015661

[123] Voora D , Coles A, Lee KL, Hoffmann U, Wingrove JA, Rhees B, Huang L, Daniels SE, Monane M, Rosenberg S, Shah SH, Kraus WE, Ginsburg GS, Douglas PS. An age- and sex-specific gene expression score is associated with revascularization and coronary artery disease: insights from the Prospective Multicenter Imaging Study for Evaluation of Chest Pain (PROMISE) trial. Am Heart J 2017;184:133–140. 28224927 10.1016/j.ahj.2016.11.004

[124] Ladapo JA , Budoff M, Sharp D, Zapien M, Huang L, Maniet B, Herman L, Monane M. Clinical utility of a precision medicine test evaluating outpatients with suspected obstructive coronary artery disease. Am J Med 2017;130:482 e411–482 e417. 10.1016/j.amjmed.2016.11.021 27993573

[125] Badimon L , Devaux Y. Transcriptomics research to improve cardiovascular healthcare. Eur Heart J 2020;41:3296–3298. 33033839 10.1093/eurheartj/ehaa237

[126] Badimon L , Robinson EL, Jusic A, Carpusca I, de Windt LJ, Emanueli C, Ferdinandy P, Gu W, Gyöngyösi M, Hackl M, Karaduzovic-Hadziabdic K, Lustrek M, Martelli F, Nham E, Potočnjak I, Satagopam V, Schneider R, Thum T, Devaux Y. Cardiovascular RNA markers and artificial intelligence may improve covid-19 outcome: position paper from the EU-CardioRNA cost action CA17129. Cardiovasc Res 2021;117:1823. 33839767 10.1093/cvr/cvab094 PMC8083253

